# Rising CO_2_ Increases Modeled Leaf Assimilation in Anatolian Forest Trees, but Thermal Threshold Exceedance Also Rises

**DOI:** 10.3390/plants15213438

**Published:** 2026-11-04

**Authors:** Emre Yazar, Bülent Akgün, Emre Babur

**Affiliations:** 1 Department of Bioengineering and Sciences, Graduate Education Institute, Kahramanmaraş Sütçü İmam University, Kahramanmaraş 46050, Türkiye; emre.yazar6@gmail.com; 2 Department of Forest Engineering, Faculty of Forestry, Kahramanmaraş Sütçü İmam University, Kahramanmaraş 46050, Türkiye

**Keywords:** photorespiration, Farquhar–von Caemmerer–Berry model, plant hydraulics, leaf energy balance, thermal safety margin, CO_2_ fertilization, ISIMIP3b, attribution, Anatolia, SSP scenarios

## Abstract

Rising atmospheric CO_2_ suppresses photorespiration, whereas warming and drying can limit leaf carbon gain. We coupled a Farquhar–von Caemmerer–Berry photosynthesis model with soil–plant hydraulics and a leaf energy balance, assigning photosystem II thermal thresholds based on leaf habit. Using ISIMIP3b forcing from five CMIP6 models, we simulated eight tree species across 27 species–site combinations at six locations in Türkiye, comparing 2020–2030 with 2070–2080 under SSP5-8.5. With soil water tracked year-round, the mean seasonal net assimilation increased by 106.5 gC m^−2^ of leaf area (95% CI: +43 to +167), with declines in 11 of 135 cells; with soil moisture reset each season, it increased by 130.3 gC m^−2^ (95% CI: +76 to +187). In the reset configuration, the contributions were +219.5 gC m^−2^ from CO_2_, −66.7 from temperature and the associated vapor pressure deficit (VPD), −38.8 from precipitation, and −2.8 from radiation (residual: +19.1). Species and sites accounted for 81% of the fitted variance, compared to 3.1% for GCMs. Days with peak leaf temperature exceeding assigned thermal thresholds increased from 1.5 to 12.2 per season, primarily in broadleaved species. This number depends on the assumed thresholds (16.9 days with a common threshold of 44 °C), energy balance parameters, and stomatal behavior, and does not quantify tissue damage. Within the tested configurations, higher modeled leaf assimilation coexisted with more frequent threshold exceedance. Neither outcome alone establishes stand productivity or forest resilience.

## 1. Introduction

Rising CO_2_ levels and increasing water stress have opposing effects on leaf carbon gain in Mediterranean and Anatolian forests. In 2015–2024, land ecosystems took up about 21% of anthropogenic CO_2_ emissions and the ocean took up about 29% [1]. The persistence of the land share depends on how leaf carbon gain responds to rising CO_2_, warming, and drying. Higher CO_2_ levels in the atmosphere suppress Rubisco oxygenation and reduce photorespiration. Walker et al. [2] estimated that photorespiration accounts for 36% of the reduction in soybean production at current CO_2_ concentrations, declining to 16% at end-of-century concentrations under RCP8.5. While these crop estimates illustrate the potential importance of photorespiration, they do not quantify its effect in forests. Warming and drying increase evaporative demand, which can induce hydraulic stress and stomatal closure [3,4]. Evaluating the balance between these responses requires a model that couples photosynthesis and water supply.

Photosynthesis and hydraulics are interdependent. Hydraulic stress promotes stomatal closure, which lowers the concentration of intercellular CO_2_ (C_i_) and limits assimilation. Sperry et al. [5] modeled these coupled responses in U.S. forests under RCP scenarios. Without acclimation, the rise in CO_2_ relative to warming was large enough to avoid chronic hydraulic stress in 55% of their climate projections. When leaf area, photosynthetic capacity, and stand density were allowed to acclimate to the new CO_2_ and climate, net primary productivity and biomass increased in 71% of the projections. However, this acclimation did not compensate for the reductions in stand net primary productivity caused by stronger warming. Differences from our leaf-level results are discussed in Section 3.1. MEDFATE [6] couples photosynthesis, soil–plant hydraulics, pressure-volume relationships, and energy balances for sunlit and shaded leaves. Both models represent canopies and stands, which our single leaf does not. What this study adds is a different use of a coupled leaf model. First, it attributes the projected change in leaf assimilation to CO_2_, temperature and vapor pressure deficit, precipitation, and radiation, with a leave-one-out and a full factorial design, and it splits the CO_2_ contribution into its direct and water-mediated parts. Second, it assesses carbon gain and exposure to photosystem II thresholds from the same leaf energy balance. Third, it applies these analyses to Anatolian tree species at sites from the Mediterranean to the Black Sea, with bias-adjusted forcing from five GCMs. Species distribution models for forest trees in Türkiye address habitat suitability rather than leaf carbon uptake [7,8,9,10]. An earlier assessment of Türkiye [11] used Monte Carlo simulations of expert-elicited stress fractions to estimate regional net primary productivity losses. This study calculates leaf-level flux responses from a process model, and its outputs are not directly comparable to those regional productivity estimates.

**Figure 1:**
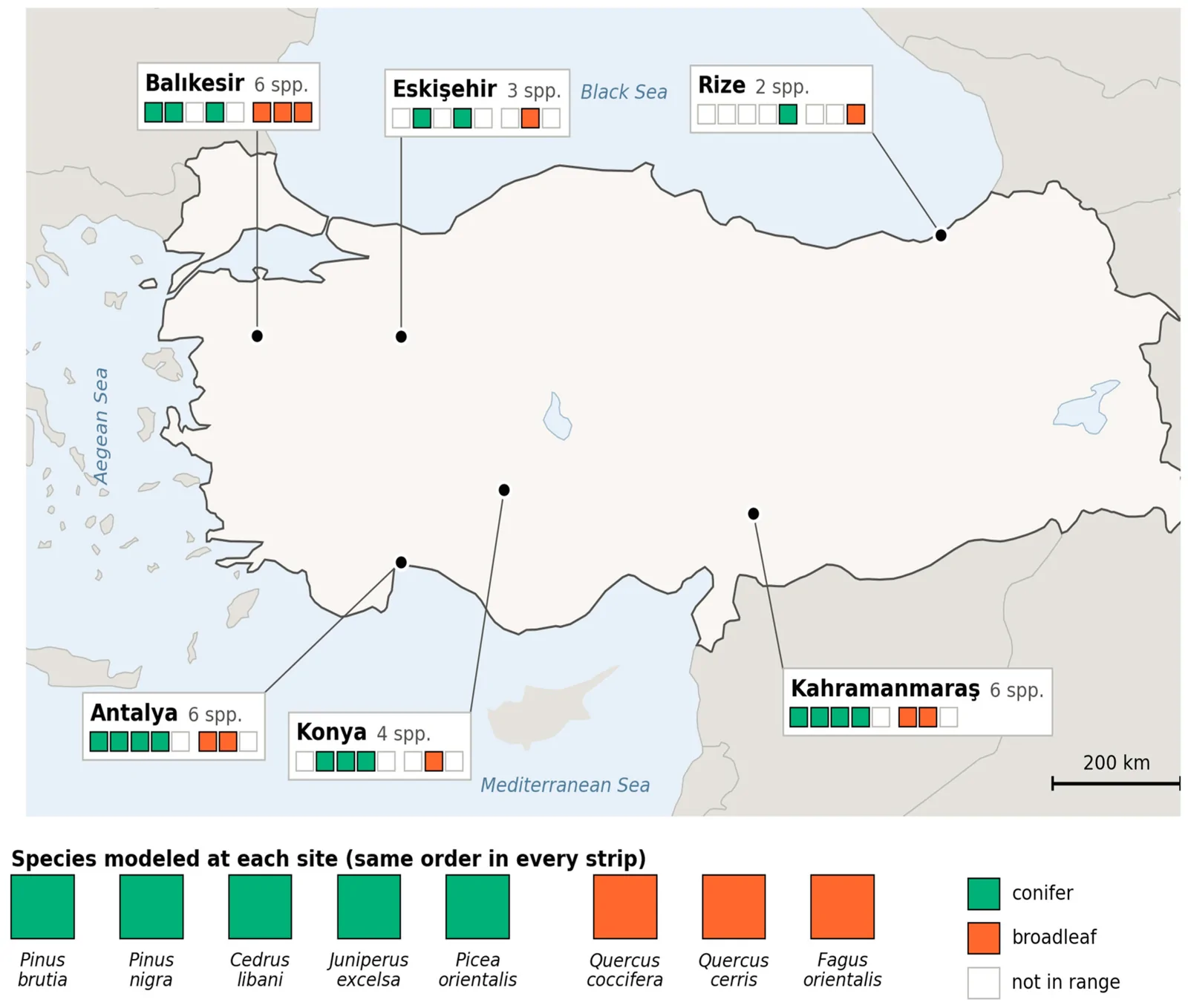
The study sites and the species that are modeled at each site. Each strip lists the eight species in the same order as in the key below the map: first the five conifers and then the three broadleaved species. A filled square means that the species is within its known natural range at that site (Section 4.4); an empty square shows that the species is absent or is present only in plantations there and has therefore not been modeled at that site. Throughout, *Pinus nigra* refers to subsp. *pallasiana*. The site markers correspond to the ISIMIP3b extraction points. The country boundaries are from Natural Earth at a scale of 1:50,000,000 (public domain).

Leaf temperature provides an additional dimension of climate exposure. In a five-year experiment on warming and soil moisture, Kullberg et al. [12] discovered that heat–drought interactions in *Fagus sylvatica* and *Quercus pubescens* occurred through leaf temperature. With warming alone, when soil water was ample, both species acclimated their leaf thermoregulation: leaf temperature stayed stable, and thermal safety margins, the difference between the critical temperature of photosystem II and the maximum leaf temperature, remained positive. However, when combined with chronic drought, warming reduced evaporative cooling and exposed leaves to temperatures that impaired photosystem II function. Critical temperatures did not differ among treatments. We developed a leaf model that couples photosynthesis with soil–plant hydraulics, stomatal closure at the turgor loss point, and a leaf energy balance; its electron-transport-limited rate follows from an explicit ATP/NADPH budget (Section 4.1). Thermal thresholds were assigned based on leaf habit. The deciduous threshold was derived from measurements taken from other species, and the higher thresholds assigned to sclerophyll and conifer leaves are assumptions, which we test with a common threshold and with group-specific shifts (Section 2.10). We applied the model to eight Anatolian tree species at six locations in Türkiye (Figure 1), using daily ISIMIP3b bias-adjusted forcing from five CMIP6 GCMs under the SSP5-8.5 scenario. SSP1-2.6 was simulated with the five GCMs for the net change and with one GCM for the attribution. We tested three hypotheses. (H1) Because the year-to-year variation in seasonal assimilation is of the same order as the projected change, single-year time slices misrepresent the change and the agreement among GCMs, so multi-year windows are needed. (H2) At the leaf level, the CO_2_ contribution exceeds the combined costs of warming and drying, as expected from the suppression of photorespiration and from free-air CO_2_ enrichment (FACE) experiments [13], and part of it is mediated by soil water saved through lower stomatal conductance. (H3) Exceedance of photosystem II thresholds nevertheless increases, more in broadleaved than in needle-leaved species because broadleaves are less coupled to the air, so that carbon gain and thermal exposure give different assessments of the same leaves.

## 2. Results

### 2.1. Single-Year Periods and 11-Year Windows

**Figure 2:**
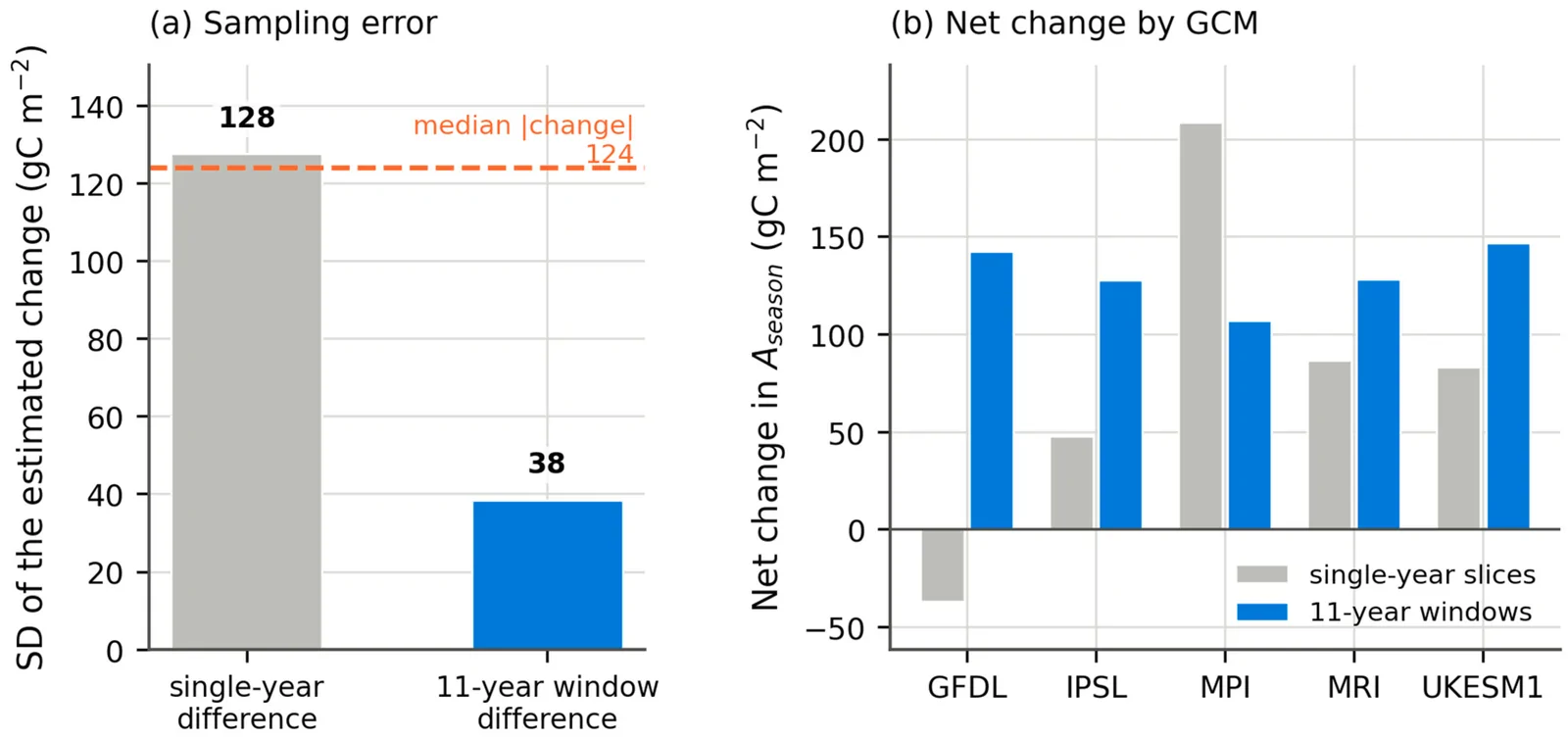
A comparison of the single-year time slices with the 11-year windows. (**a**) The standard deviation of the estimated change in *A*_season_ when comparing two single years and when comparing two 11-year windows (medians over 135 cells); the dashed line indicates the median absolute projected change. (**b**) The net change in *A*_season_ under SSP5-8.5 for each GCM, calculated from the single-year slices and from the 11-year windows on the same 27 cells per GCM.

The year-to-year variation in the model was about equal to the projected change. One cell is defined as one species at one location using one GCM. The SSP5-8.5 ensemble consists of 135 cells (27 in-range species–site combinations multiplied by five GCMs). The assimilation values are all seasonal net assimilation per unit leaf area (*A*_season_, in gC m^−2^ per season). The median standard deviation of *A*_season_ between years within a given window was 68 gC m^−2^ from 2020 to 2030 and 103 gC m^−2^ from 2070 to 2080. This means that the year-to-year variability in the 2070s was 1.5 times greater than in the 2020s. Consequently, the difference between two individual years had a median standard deviation of 128 gC m^−2^, which is nearly equal to the median absolute projected change of 124 gC m^−2^ (Figure 2a). When the data are averaged over 11-year windows, the standard deviation is reduced to 38 gC m^−2^.

Single-year sampling affected both the precision and the sign of the result. Using time periods of one year, the five GCMs produced different results for the projected change. Their estimates differed by up to 246 gC m^−2^. However, with 11-year windows on the same cells, all five GCMs predicted an increase, and the range decreased to 40 gC m^−2^ (Figure 2b). MPI-ESM1-2-HR showed the greatest increase in single years and the smallest increase in 11-year windows. It also had the highest precipitation cost among the five GCMs, at −69 gC m^−2^ (Table 1). Therefore, the apparent disagreement among the GCMs was mainly due to sampling error (Section 3.2). All subsequent results are based on 11-year windows (2020–2030 and 2070–2080), unless otherwise stated.

### 2.2. Attribution to Forcing Channels

The baseline year differs from the scenario year in four climate drivers or forcing channels: atmospheric CO_2_, temperature (along with the associated vapor pressure deficit), precipitation, and radiation. We estimated each channel’s contribution by holding that channel at its baseline value while the other three followed the scenario (see Section 4.6). Since the coupled response is nonlinear, the individual “leave-one-out” contributions are not additive. Each contribution is therefore conditional on the model and on the other three channels: it measures the modeled response to the change in one channel when the others have changed, not a separable physical effect of that channel (Section 2.10 compares three ways of measuring it). The difference between the projected change and the sum of the four contributions is given as a residual. Since every season begins with the same relative soil moisture (see Section 4.2), the precipitation channel only includes changes in precipitation during the growing season. Section 2.9 examines changes in winter recharge. The seasonal-reset configuration is used for attribution to keep initial soil moisture fixed and isolate within-season forcing responses. This configuration is an experimental control rather than a realistic assumption about winter recharge. Therefore, the year-round results must accompany the interpretation of the reference attribution.

**Figure 3:**
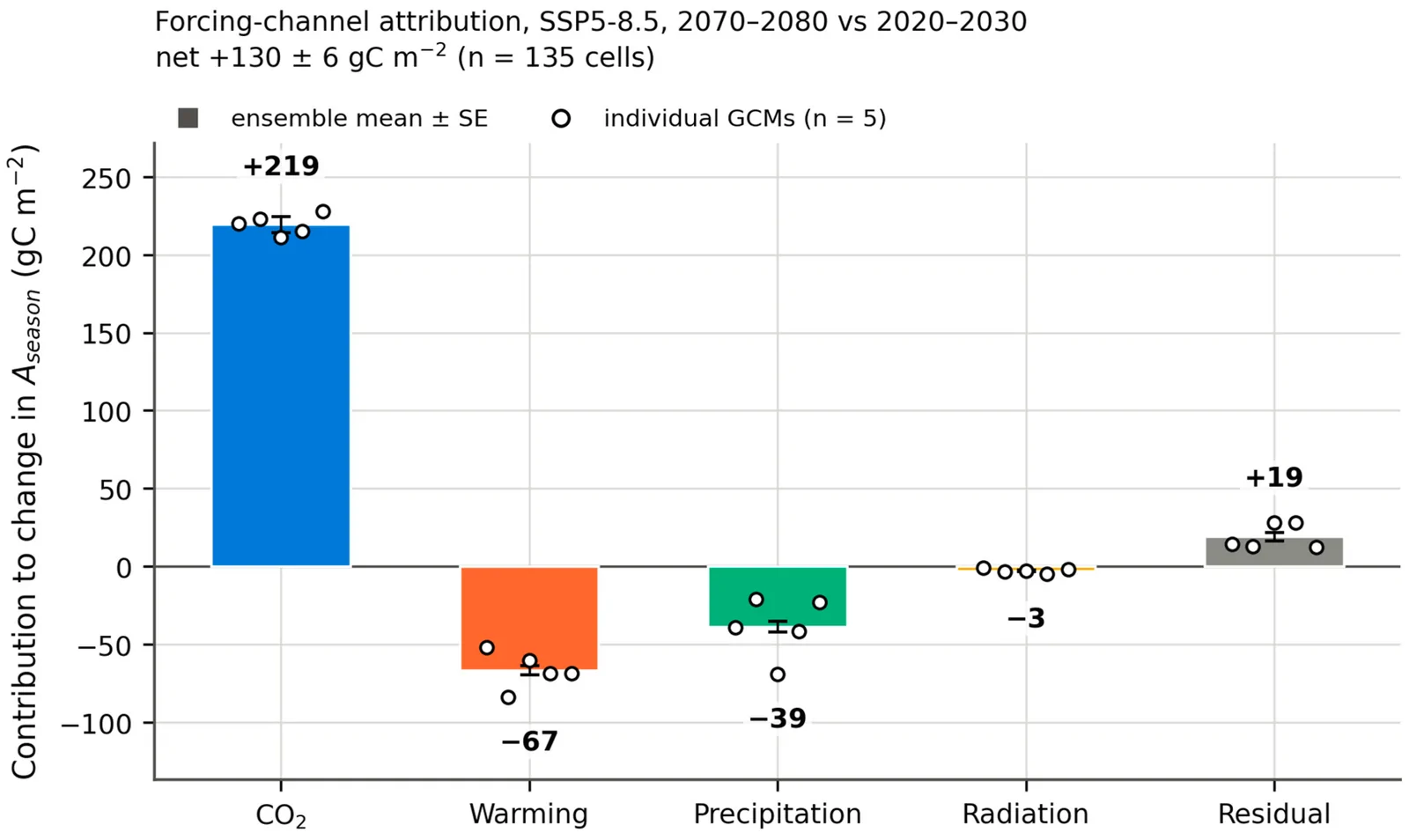
The contribution of each forcing channel to the change in *A*_season_ between 2020–2030 and 2070–2080 is shown under SSP5-8.5 (135 cells: 27 species–site combinations × five GCMs). The bars display the ensemble mean ± standard error; the open circles represent the individual GCMs. The residual refers to the portion of the projected change that cannot be attributed to any one channel.

In the SSP5-8.5 scenario, each of the five GCMs showed an increase in *A_season_* (see Table 1 and Figure 2b). The ensemble mean rose from 446.3 to 576.6 gC m^−2^ (+130.3 gC m^−2^), and individual GCMs ranged from +107 to +147 gC m^−2^. The contribution from CO_2_ (+219 gC m^−2^) was greater than the losses due to warming (−67 gC m^−2^) and those due to precipitation (−39 gC m^−2^), whereas the effect of radiation was small (−2.8 gC m^−2^; Figure 3). Photorespiration fell from 76 to 59 gC m^−2^, or from 14.6% to 9.3% of gross assimilation (defined as net assimilation plus the CO_2_ released through photorespiration), consistent with the anticipated impact of CO_2_. In Section 2.2, Section 2.3 and Section 2.5, the ± values are the standard errors (SE) across cells; they are descriptive estimates of mean precision under an independence assumption and do not quantify dispersion or total projection uncertainty. The range among the five GCMs is not a complete measure of projection uncertainty either: Section 2.6 calculates a broader interval, and Section 3.4 addresses structural uncertainty.

Two aspects of this partition are worth mentioning. First, the CO_2_ contribution was nearly the same across all GCMs (211–228 gC m^−2^, a difference of 8%). This result is expected because all GCMs specify C_a_ the same way, and differences in this contribution among models arise solely from how it interacts with each model’s climate; the small range thus serves as a consistency check for the partition. Second, the four contributions did not add up to the projected change, leaving a residual of +19 gC m^−2^ (15% of the total change). In a leave-one-out decomposition of a nonlinear system, the residual includes all higher-order terms, and its magnitude depends on the decomposition method. Therefore, we report the residual as is without interpreting it as a physical interaction. For the GFDL-ESM4 model, the residual decreased from +14.4 gC m^−2^ under the SSP5-8.5 scenario to +3.4 gC m^−2^ under the SSP1-2.6 scenario (see Table 2). This change may reflect either the size of the perturbation or the weather of the paired years. A full factorial design with year-round soil water assigns the residual to the channels with Shapley values and changes neither the signs nor the order of the four contributions in the ensemble mean (Section 2.10, Table A6). A test in which the weather was kept in sequence (Section 2.9) indicates that approximately two-thirds of the residual results from pairing the weather of different years.

### 2.3. Scenario Differences in Assimilation and Thermal Threshold Exceedance

**Figure 4:**
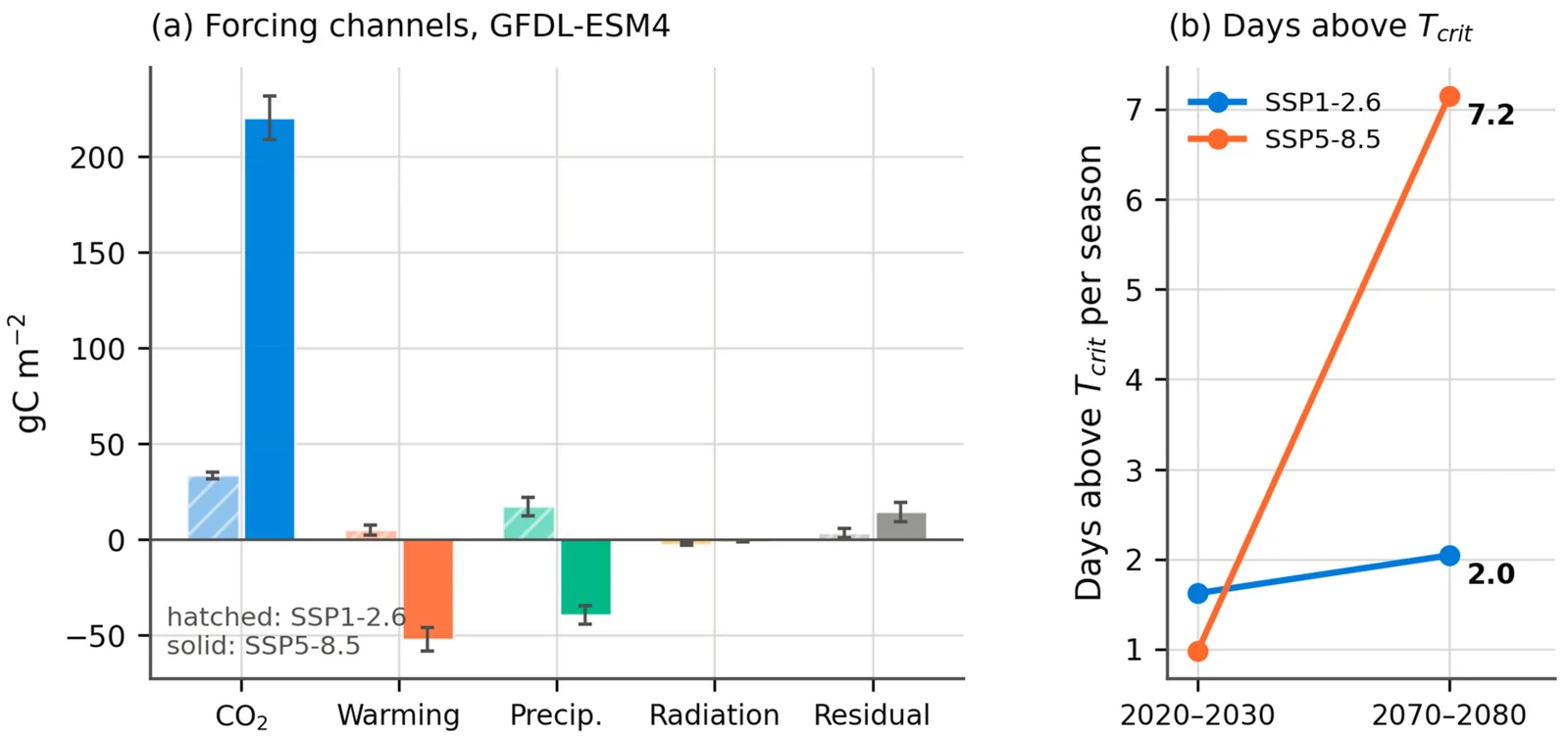
A comparison of the scenarios for GFDL-ESM4 (27 cells). (**a**) The forcing-channel contributions under SSP1-2.6 (hatched) and SSP5-8.5 (solid), mean ± SE. (**b**) The number of days per season when the peak leaf temperature exceeded *T*_crit_.

The SSP1-2.6 attribution used only the GFDL-ESM4 model, so the comparison of channel contributions between the scenarios is based on a single model and should be considered preliminary (the net change under SSP1-2.6 with all five GCMs and year-round soil water is given in Section 2.10). With GFDL-ESM4, *A*_season_ increased in both scenarios for different reasons (see Table 2 and Figure 4a). Under SSP1-2.6, C_a_ rose from 427.7 to 471.0 μmol mol^−1^, but the amount contributed by CO_2_ (+33.6 gC m^−2^) was much smaller than under SSP5-8.5. The mild warming (+5.0 gC m^−2^) and slightly wetter growing seasons (+17.2 gC m^−2^) together accounted for approximately two-thirds as much. Under SSP5-8.5, the increase in the same cells was greater (+142.4 rather than +56.9 gC m^−2^) due to the CO_2_ channel (+220.2 gC m^−2^). This more than compensated for losses resulting from warming (−52.0 gC m^−2^) and precipitation (−39.3 gC m^−2^). These losses did not occur under SSP1-2.6. However, one limitation does apply: the two scenarios start from different baseline realizations. The baselines differ by 17 gC m^−2^ in *A*_season_, which is about one-fifth of the difference between the changes in the two scenarios (85.5 gC m^−2^). They also differ by 0.6 in the number of days per season on which the peak leaf temperature exceeds the critical photosystem II temperature (*T*_crit_). Therefore, the contrast between the scenarios is more reliable for 2070s levels than for changes. With the five GCMs, we therefore compared the scenarios on their 2070s levels (Section 2.10).

The number of days above *T*_crit_ did not correspond to the carbon projection. For the GFDL-ESM4 model, the number of days rose from 1.6 to 2.0 per season under the SSP1-2.6 scenario (a 1.3-fold increase) and from 1.0 to 7.2 under the SSP5-8.5 scenario (a 7.3-fold increase; see Figure 4b). With all five GCMs and year-round soil water, the SSP1-2.6 number rose from 1.8 to 2.8 (Section 2.10). Across the five GCMs under SSP5-8.5, the number increased from 1.5 to 12.2 per season (an approximately eight-fold increase; see Table 2). Unlike the carbon result, this estimate varied considerably among GCMs. Values ranged from 5.2 to 17.5 days per season in the 2070s (a 3.4-fold range). This variation among GCMs is in addition to the parameter sensitivity described in Section 2.5. All GCMs projected an increase, though the magnitude varied from 5.6- to 11.8-fold. The difference between species groups was large and opposite to the carbon result. Days above T*_crit_* were concentrated among the three broadleaved species since their leaves are the least coupled to the air, and they have the lowest assigned T*_crit_* (Section 4.3). Without the difference in assigned thresholds, the contrast was smaller but remained: with a common threshold of 44 °C, the broadleaved species exceeded it on 31.7 days per season in the 2070s and the conifers on 8.2 (Section 2.10). In the 2070s, *Quercus cerris* had 37 days per season, *Quercus coccifera* had 21, and *Fagus orientalis* had 20. In comparison, each conifer had three or fewer days. The two species that showed the greatest carbon gains (Table 3)—*Picea orientalis* and *Juniperus excelsa*—had 0.8 and 2.2 days, respectively. The number of days also depends on the threshold itself, the behavior of stomata in heat, and the air temperature assigned to the stands (Section 2.9).

### 2.4. Attribution by Species

**Figure 5:**
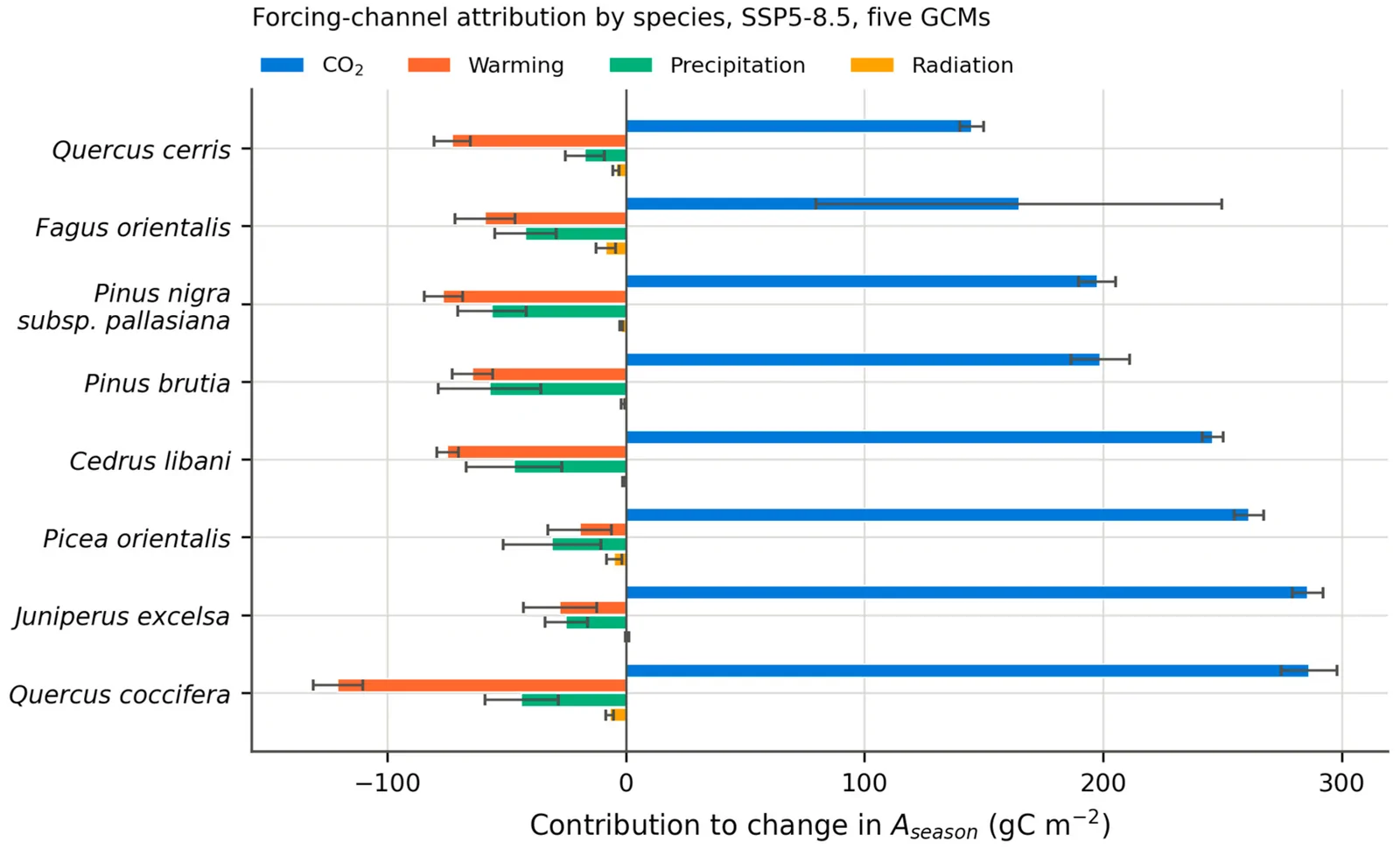
The forcing-channel attribution by species under SSP5-8.5 is shown, with the data pooled over each species’ in-range sites and involving the five GCMs and the 11 window years (mean ± SE); the error bars correspond to the standard errors listed in Table 3, which come from a model that includes site and GCM as crossed random effects.

We obtained the species-level contributions shown in Table 3 and Figure 5 by pooling the in-range sites and five GCMs for each species. The CO_2_ contribution varied from +145 gC m^−2^ for *Quercus cerris* to +286 gC m^−2^ for *Juniperus excelsa* and *Quercus coccifera*. The latter had the highest warming cost, −121 gC m^−2^. These differences require caution. In the model, species vary in their sites and trait values. Most trait values are assumed or borrowed from related species. For all eight species, rooting depth and carboxylation capacity are assumed or borrowed. For all species except *Cedrus libani*, leaf area index (LAI) is assumed or borrowed (see Appendix A). Section 2.10 shows how much the net change depends on rooting depth, LAI, carboxylation capacity, the stomatal slope, and the turgor loss point. Thus, the ranking reflects these assumptions as much as it reflects physiology. Therefore, Table 3 should be regarded as a diagnosis of the trait assumptions rather than a projection of relative risk among species. The ranking also depends on the soil water balance (Section 2.9).

The stem P_50_ value (the water potential at 50% loss of hydraulic conductivity) for Quercus coccifera was assumed to be the same as that for Q. ilex. Three sources provide the following values: −3.88 [14], −4.66 [15], and −7.13 MPa [16] (see Appendix A). In all of the aforementioned runs, the value of −7.13 MPa was used. For *Q. coccifera* itself, the compilation of Robert et al. [15] gives −6.96 MPa, within 0.2 MPa of this value. We reran the 15 cells (three sites × five GCMs, SSP5-8.5, and both windows) using the other two values. Using the default value, the net change for Q. coccifera was +153.2 gC m^−2^; using −4.66 and −3.88 MPa, the net change was +153.1 and +153.0 gC m^−2^, respectively. No channel changed by more than 0.3 gC m^−2^, and the ensemble change remained at +130 gC m^−2^ within the same range (see Section 2.6 for details). No species changed its rank in any column of Table 3 or in the net change (see Table A1). This lack of sensitivity is due to the model structure. The stomata close at the turgor loss point of −2.6 MPa, which occurs before the leaf water potential reaches any of the three P_50_ values. At that water potential, the plant’s hydraulic conductance has decreased by only 12%, 27%, or 36%, depending on the P_50_ value: −7.13, −4.66, or −3.88 MPa, respectively. For this species, therefore, the assumed turgor loss point is more important than P_50_.

### 2.5. Sensitivity to CO_2_ Acclimation and to Energy Balance Parameters

**Figure 6:**
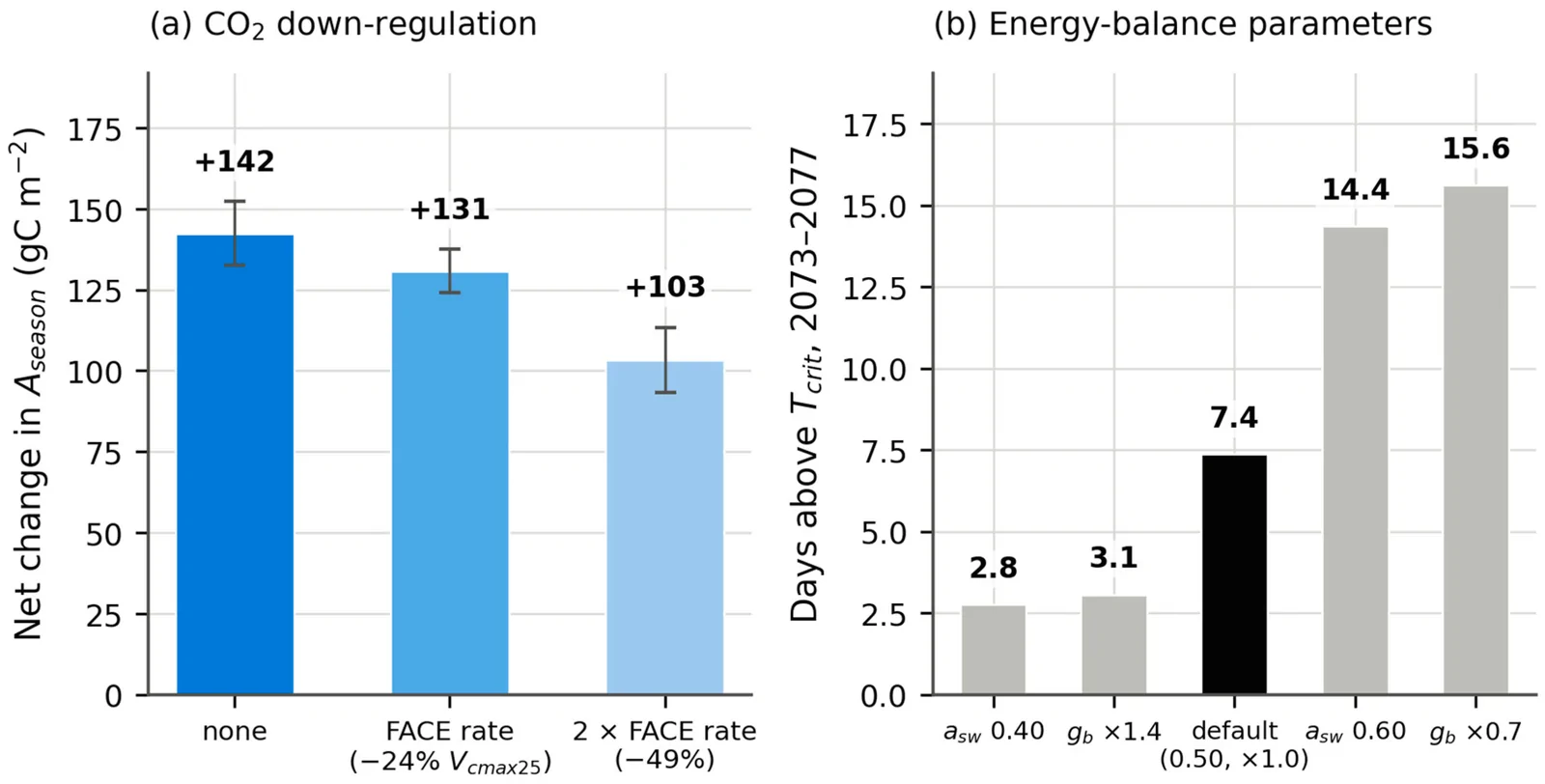
Sensitivity to CO_2_ acclimation and to energy balance parameters. (**a**) The net change in *A*_season_ without acclimation of photosynthetic capacity to growth CO_2_, with the down-regulation rate derived from the FACE experiments, and with twice that rate (GFDL-ESM4, 11-year windows, mean ± SE; the percentages are the reductions in carboxylation capacity applied at 801.7 μmol mol^−1^). (**b**) The number of days above *T*_crit_ from 2073 to 2077 over the plausible ranges of the two least-constrained energy balance parameters; the default parameterization is indicated in black.

The CO_2_ contribution is the largest term in the attribution, and the model does not include processes that would constrain it. There is no feedback from nonstructural carbohydrates, no limitation due to the sink, and no acclimation of carboxylation capacity to growth CO_2_. Therefore, we used the down-regulation described in the meta-analysis of free-air CO_2_ enrichment (FACE) experiments by Ainsworth and Long [13]: a mean decrease of 13% in the maximum carboxylation rate (*V*_cmax_) and 5% in the maximum electron transport rate (*J*_max_) at elevated CO_2_ levels of 475–600 μmol mol^−1^, about 200 μmol mol^−1^ above ambient. These means combine all C_3_ functional groups. Trees showed smaller decreases, about 6% in *V*_cmax_ [17] with no significant decrease in *J*_max_ [13], and no down-regulation was found in mature *Fagus*, *Quercus*, and *Carpinus* trees after eight years at about 550 μmol mol^−1^ [18] or in mature *Quercus pubescens* growing near natural CO_2_ springs [19]. Our rate is therefore about twice the FACE rate for trees. The reduction was scaled per 100 μmol mol^−1^ above the baseline concentration (Section 4.7), which extrapolates it linearly beyond the FACE range to 801.7 μmol mol^−1^. This reduced the ensemble gain by 8%, from +130.3 to +120.0 gC m^−2^, as well as the GFDL-ESM4 gain by 8%, from +142.4 to +130.8 gC m^−2^. Doubling the down-regulation decreased the GFDL-ESM4 gain by 27%, to +103.3 gC m^−2^ (Figure 6a); this doubled rate (a 49% lower *V*_cmax25_ at 801.7 μmol mol^−1^) is about four times the FACE rate for trees. Therefore, the sign of the result was robust with respect to acclimation of the photosynthetic capacity. However, this test considers only one aspect of the uncertainty related to the CO_2_ effect. It did not include progressive nitrogen limitation and sink feedbacks, which would also reduce the gain. In forest FACE experiments, nutrient limitation acted mainly on the conversion of the additional carbon into growth: the enhancement of net primary productivity of a sweetgum stand declined from 24% to 9% as nitrogen availability fell [20], and a 12% increase in the gross primary productivity of a mature eucalypt forest did not increase ecosystem carbon storage [21]. Leaf-level photosynthetic gains persisted in these experiments [22]. Within the tested range, nutrient or sink limitation that lowers photosynthetic capacity would therefore reduce the magnitude of the modeled leaf-level gain without reversing its sign, but it could remove the corresponding gain in stand growth, which a leaf model does not represent. Section 3.1 compares the leaf-level gain with the gains measured from leaf to stand in forest FACE experiments.

One reason for this modest effect is evident in the water budget. As down-regulation increased, seasonal transpiration decreased from 7337 to 7066 and 6606 mol m^−2^ (GFDL-ESM4, 2070–2080). The number of days when stomata were effectively closed also decreased from 26.8 to 23.3 and 17.2. Since leaves with lower photosynthetic capacity transpire less, soil water lasts longer, extending the productive season further into summer. However, this water conservation only partially compensates for the capacity loss.

The second weakly constrained model component is the leaf energy balance. The two least constrained parameters—namely, the shortwave absorptance (*a*_sw_ = 0.50) and the boundary-layer conductance (*g*_b_), which is assigned according to leaf habit—both influence the difference in temperature between the leaf and the air. Over their plausible ranges (GFDL-ESM4, 27 cells; Section 4.7), the mean seasonal maximum of the leaf–air temperature difference in the period 2073–2077 varied from 3.74 to 6.98 °C around the default value of 5.13 °C, and the number of days above *T*_crit_ varied from 2.76 to 15.61 around the default of 7.36, representing a 5.7-fold range (Figure 6b, Table 4). The absolute number of days above *T*_crit_ is thus uncertain and should therefore be given together with this range. Yet in every parameter variant (in each case only one parameter was changed) there was a large increase from the 2020s to the 2070s (a 3.7- to 31-fold increase), while the net change in *A*_season_ remained between +150.0 and +162.2 gC m^−2^ (the default being +156.2 gC m^−2^; a change from −4.0% to +3.8%). The carbon result is therefore not sensitive to the energy balance parameterization, although the number of days above *T*_crit_ depends strongly on it in magnitude but not in direction. These tests use five-year windows (2023–2027 and 2073–2077), so their default values (7.36 days and a net change of +156.2 gC m^−2^) differ from the 11-year values for GFDL-ESM4 in Table 2 (7.2 days and +142.4 gC m^−2^).

### 2.6. Uncertainty in the Ensemble Mean Change

The reported descriptive standard errors assume independence across cells, but species share sites, and GCMs share a forcing protocol and bias-adjustment method. Therefore, we fitted a crossed random-effects model with species, site, and GCM effects using restricted maximum likelihood (REML; see Section 4.9). The resulting interval concerns the ensemble mean change under the specified model, not the range of possible changes in individual forests or total projection uncertainty.

The standard error associated with the generalized least squares (GLS) estimate of the net change was 28.2 gC m^−2^, compared to the naive standard error of 5.7 gC m^−2^ (a 5.0-fold increase). The 95% confidence interval ranged from +76 to +187 gC m^−2^ and remained positive. However, this interval only refers to the sampled species, sites, and GCMs within the specified model structure. The five GCMs were bias-adjusted to the same observation-based dataset and share the prescribed CO_2_ pathway, so they are not independent samples of possible climates, and the interval is also conditional on this forcing protocol. It does not account for uncertainty in mostly assumed species traits or canopy representation. Section 2.9 provides the interval obtained when soil water is simulated year-round.

In the fitted variance partition (Table 5), species accounted for 50.2%, sites for 30.7%, and GCMs for 3.1%. These components describe the heterogeneity within the sampled ensemble. The interval calculated from the five GCM means (+111 to +150 gC m^−2^, with four degrees of freedom) was narrower than the crossed random-effects interval. Therefore, GCM variation alone did not capture the variability associated with species and site selection in this design. The partition differed among forcing channels. GCM and unexplained variance were small for CO_2_ (0.3% and 1%), which is consistent with shared CO_2_ forcing. However, 63% of the variance in the precipitation channel was not explained by the three random effects. This residual may include species–site–GCM interactions and weather variability that the fitted model does not distinguish.

The approximate, model-based, 95% intervals retained the signs of all four channel means: CO_2_, +134 to +304 gC m^−2^; warming, −89 to −39; precipitation, −62 to −18; and radiation, −6.7 to −0.3. For net change, species and site heterogeneity contributed more to the fitted variance than GCM differences. However, these intervals do not account for uncertainty in trait values, omitted processes, or canopy structure.

### 2.7. Comparisons with Published Measurements

We did not test the model against season-long leaf flux data for these species because suitable data is scarce. The comparisons assembled here do not include independent seasonal leaf-flux observations for all modeled species or sites. For this reason, we used only published stand and leaf measurements as rough benchmarks, acknowledging the differences in spatial and temporal scales. To compare modeled leaf fluxes with stand measurements, we multiplied the fluxes by each species’ LAI to express them on a per-unit-ground-area basis. Since the model simulates a single sunlit leaf and does not account for self-shading or within-canopy humidity gradients, this approach assumes that all leaves in the canopy behave as if they were sunlit leaves at the top of the canopy. Thus, it is an upper limit, and the amount by which it exceeds the measured values shows the impact of missing canopy integration. Scaling by the sunlit leaf area only, [1 − exp(−0.5 LAI)]/0.5 (Section 4.7), gives a lower bound, because the shaded leaves, which fix carbon and transpire at lower rates, are then omitted; a canopy with sunlit and shaded leaves lies between the two bounds.

*Pinus brutia* is the only species for which we compiled both carbon and water benchmarks (see Table 6). The model estimated 884 gC m^−2^ per season, compared with measured and modeled gross primary productivity (GPP) of 445–880 gC m^−2^ yr^−1^ for this species along a post-fire chronosequence and an elevation gradient [23]. Modeled transpiration amounted to 278 mm per season, compared to 109 mm over a nine-month dry period and 296 mm over a 19-month sap-flow record in a *P. brutia* stand in Athalassa, Cyprus [24]. The modeled carbon flux is at or slightly above the upper end of the published range. The modeled transpiration rate exceeds the observed rates, both of which align with the assumption of sunlit leaves. Scaled by the sunlit leaf area of 1.26, the values were 559 gC m^−2^ and 176 mm, so the published carbon range overlaps the interval between the two bounds, and the sap-flow transpiration is closer to the lower one. The net ecosystem productivity (NEP) of five native Turkish conifer stands, four of which are species examined in this study, was 2.0 ± 1.1 Mg C ha^−1^ yr^−1^ [25]. Because NEP is calculated after accounting for all respiration, however, it only provides a lower bound for comparison.

Water-use efficiency offers an additional plausibility check, even though agreement in this ratio does not prove that the assimilation and transpiration values are correct. The average ratio of assimilation to transpiration (A/E) in the model was 4.85 mmol CO_2_ mol^−1^ H_2_O across the different species, with the ratio ranging from 2.8 to 7.7 mmol mol^−1^, which is within the normal range for C_3_ tree leaves as inferred from the instantaneous rates given for *P. brutia*, *P. nigra* subsp. *pallasiana* [26] and *Cedrus libani* [27]. This ratio represents the amount of carbon fixed per unit of water transpired; it is not the intrinsic water-use efficiency (A/g_s_, where g_s_ is stomatal conductance).

Only leaf temperature can be compared for pines. For *Pinus brutia* and *Pinus nigra*, the mean maximum seasonal difference between leaf and air temperature, as modeled for 2020–2030, was 3.8 °C. Müller et al. [28] recorded mean differences of 2.4 ± 0.6 °C for drought-exposed trees and 3.0 ± 0.8 °C for irrigated trees in a semi-arid pine forest when shortwave radiation was above 600 W/m^2^, with differences below 3 °C on midsummer days in both cases. Since a seasonal maximum is expected to exceed the conditional mean, the modeled value is plausible, even though the two quantities are not directly comparable. For the three broadleaved species, the modeled seasonal maximum was 6.8–8.8 °C; no corresponding measurements were available. These species have a high number of days above *T*_crit_ (see Section 2.3), and their thermal projection is based on peak leaf temperature; therefore, the projection is least constrained in this case. Together with the 5.7-fold range resulting from the energy balance parameters (Section 2.5), it can be concluded that only the direction of the change is robust. Under SSP5-8.5, the increase was at least 3.7-fold in every energy balance variant (GFDL-ESM4) and GCM; however, the actual magnitude is uncertain. The carbon projections do not rely on this component (Section 2.5).

The upper-bound comparison is not very informative when the LAI is high; for example, scaling the leaf flux of *Picea orientalis* by its LAI of 5.0 yields 3687 gC m^−2^ per season per unit ground area, a figure which is unreasonably high for a stand; similarly, *Fagus orientalis* (with an LAI of 5.5 and 1736 gC m^−2^) and *Quercus coccifera* (with an LAI of 3.5 and 1576 gC m^−2^) also produce high values; scaled by the sunlit leaf area, the three species give 1354, 591, and 744 gC m^−2^. In these cases, we keep the leaf-level results, but the ground-area figures in Table 6 should be treated only as arithmetic upper limits.

Taken together, the comparisons give independent but indirect support to two components only: the magnitudes of the carbon and water fluxes of *Pinus brutia*, which lie within or near the bounds set by the stand measurements, and the leaf–air temperature difference of the pines. The water-use efficiency checks only the ratio of two modeled fluxes, and the quantum yield (Section 4.1) checks the energy budget rather than field fluxes. No comparison constrains the leaf temperatures of the broadleaved species, the thermal thresholds, the projected changes, the attribution, or the species ranking, and we do not regard any of these comparisons as validation.

**Table 6:** Modeled leaf fluxes for 2020–2030 under SSP5-8.5; the published values used as benchmarks are listed below the table. A is seasonal net assimilation and E is seasonal transpiration. The ground-area values are obtained by multiplying the leaf fluxes by the species’ LAI; they represent upper bounds, not canopy simulations (see the text for details).

Species	LAI	A Leaf (gC m^−2^)	A × LAI (gC m^−2^)	E Leaf (mm)	E × LAI (mm)	A/E (mmol mol^−1^)
*Pinus brutia*	2.0	442	884	139	278	4.77
*Pinus nigra* subsp. *pallasiana*	2.5	413	1032	130	325	4.76
*Cedrus libani*	2.1	500	1050	162	341	4.62
*Juniperus excelsa*	1.5	700	1051	213	320	4.92
*Quercus coccifera*	3.5	450	1576	157	551	4.29
*Quercus cerris*	4.0	188	751	102	409	2.75
*Fagus orientalis*	5.5	316	1736	95	520	5.01
*Picea orientalis*	5.0	737	3687	144	719	7.70
species mean	3.3	468	1471	143	433	4.85

The published values include a stand GPP for *Pinus brutia* of 445–880 gC m^−2^ yr^−1^, with an average LAI of 1.96 [23]; a net ecosystem productivity for five conifer stands in the eastern Mediterranean region of Türkiye of 2.0 ± 1.1 Mg C ha^−1^ yr^−1^ [25]; sap-flow transpiration from a *P. brutia* stand at Athalassa in Cyprus (with a canopy LAI of 4.4) amounting to 109 mm over a period of nine months in the presence of 175 mm of rain and 296 mm over the entire record [24]; a leaf–air temperature difference in a semi-arid pine forest when shortwave radiation exceeds 600 W m^−2^, being 2.4 ± 0.6 °C (in the case of drought exposure) and 3.0 ± 0.8 °C (when irrigated) [28]; and a leaf A/E for C_3_ trees of 2–8 mmol CO_2_ mol^−1^ H_2_O, as inferred from the instantaneous rates given in [26,27].

### 2.8. Sensitivity to Baseline Growing Season and Soil Assumptions

The changes reported above compare the late window of each scenario with the early window of the same scenario, so the two scenarios are measured against two different realizations of the 2020s. In ISIMIP3b, both pathways start from the same historical simulation in 2015, so 2005–2014 is the last decade common to both. With this common baseline, the ensemble net change rose from +130.3 to +153.3 gC m^−2^ (see Table 7), mainly because the time span is longer (6.55 instead of 5.0 decades between the window midpoints); per decade, the estimate from the common baseline is 10% lower (+23.4 against +26.1 gC m^−2^ per decade). The scenario contrast also changed. For GFDL-ESM4, the difference between the SSP5-8.5 and SSP1-2.6 changes was 85.5 gC m^−2^ when using each scenario’s individual baseline and 102.7 gC m^−2^ when using the common baseline, which is 1.2 times as large. Therefore, the scenario-specific baselines absorbed about 17% of the difference between scenarios obtained with the common baseline. With the common baseline, the 95% interval for the ensemble change was between +86 and +225 gC m^−2^, and the share of variance attributable to the GCMs increased from 3.1% to 4.9%.

Two other assumptions relate to the growing season and the soil. Growing seasons are defined as fixed calendar periods, so the model cannot account for longer seasons due to warming, and all six sites have the same soil texture (sandy loam). With GFDL-ESM4 and five-year windows, extending the growing season by 15 days raised the net change by 4.6 gC m^−2^ (+3.0%), while shortening it by 15 days lowered it by 5.1 gC m^−2^ (−3.3%). Replacing the soil with a finer type (loam) reduced the net change by 10.9 gC m^−2^ (−7.0%), and using a coarser type (loamy sand) increased it by 5.1 gC m^−2^ (+3.3%; see Table 7). The ensemble mean remained positive in these tests; their small effects do not rule out sensitivity to other soils or phenological responses. The soil result is counterintuitive: finer soil is more stressful because plants respond to matric potential rather than water content, and at the same relative saturation, loam retains its greater water store about ten times more tightly than loamy sand. When soil water was simulated year-round, so that each soil started the season with the water remaining after its own winter, the same two soils shifted the net change by −5.1 and +1.9 gC m^−2^ relative to sandy loam (Section 2.9).

### 2.9. Tests of the Soil Water Balance, the Attribution Method, and Threshold Exceedance

**Figure 7:**
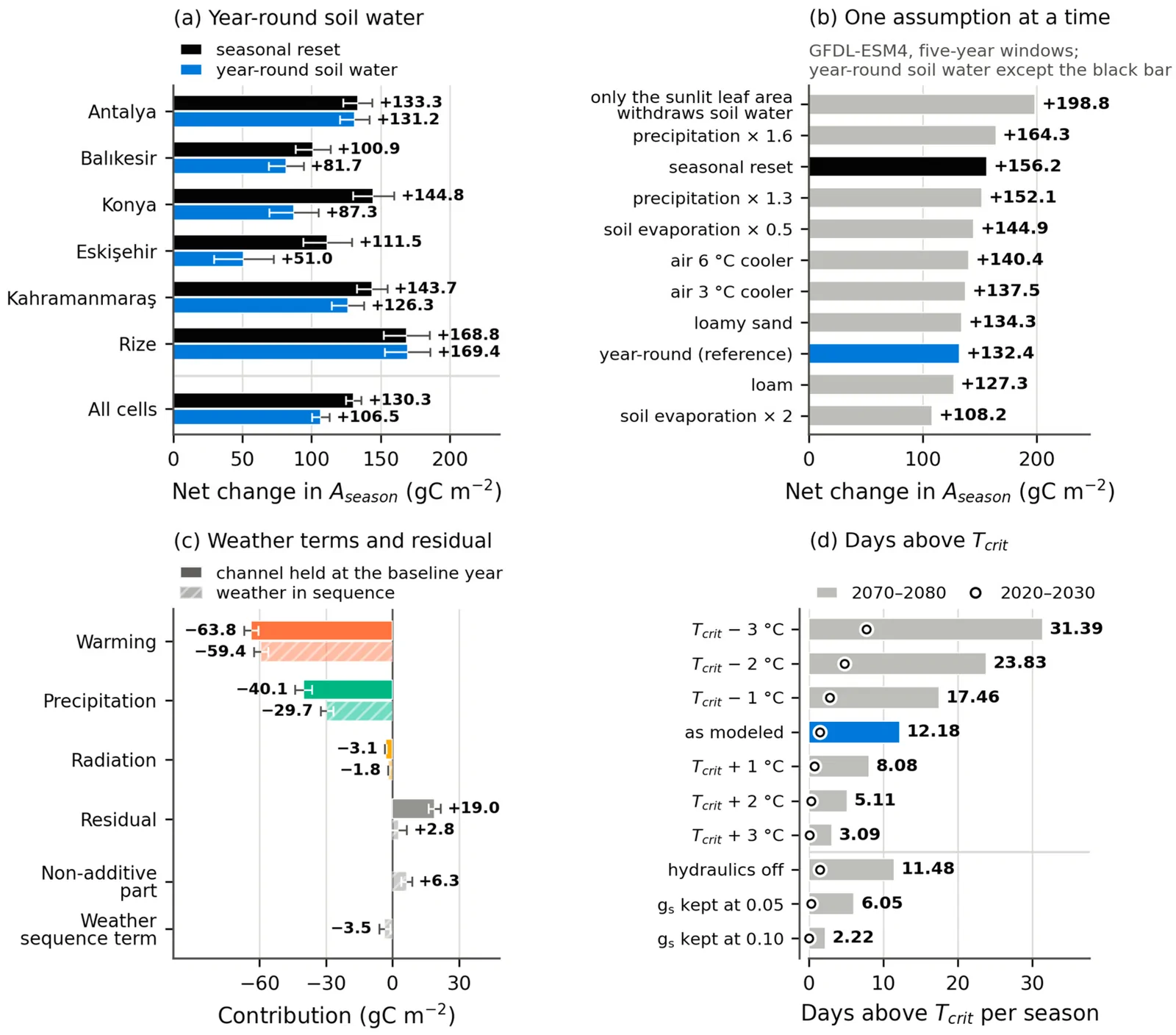
Tests of the soil water balance, the attribution method, and the number of days above *T*_crit_ under SSP5-8.5. (**a**) The net change in *A*_season_ with the seasonal reset (relative soil moisture set to 0.60 at the start of each season) and with year-round soil water, by site and for all 135 cells (five GCMs, 11-year windows, mean ± SE across cells; Table A2). (**b**) The net change when one assumption is changed at a time (GFDL-ESM4, 27 cells, five-year windows). All bars are for year-round soil water except the black one; five variants that shifted the net change by 0.2 gC m^−2^ or less are not shown (Table A3). (**c**) The three weather terms and the residual for 2073–2077, with the channel held at the paired baseline year (solid) and with the weather in sequence, that is, with the monthly mean change in the channel removed from the weather of 2073–2077 (hatched); five GCMs, seasonal reset, mean ± SE across 135 cells. With the weather in sequence, the residual is the sum of the non-additive part of the four mean changes and the weather sequence term (Table A4). (**d**) The number of days above *T*_crit_ per season in 2070–2080 (bars) and 2020–2030 (circles): as modeled with year-round soil water (blue); with the threshold shifted by 1–3 °C or for a leaf that keeps a stomatal conductance (g_s_) of 0.05 or 0.10 mol m^−2^ s^−1^ under the same forcing; and with soil–plant hydraulics switched off (five GCMs, 135 cells; Table A5, of which two rows are not shown).

In the above-reported runs, relative soil moisture (the fraction of pore space filled with water) is reset to 0.60 at the start of each season (seasonal reset; Section 4.2). Thus, a change in winter recharge cannot affect the following season. We repeated the runs for both windows, carrying soil water from each season into the next through the winter after two spin-up years (year-round soil water, Section 4.7). The results of this test and the subsequent tests are presented in Figure 7 and Appendix B. The ensemble net change decreased from +130.3 to +106.5 gC m^−2^ (−18%; see Figure 7a and Table A2), and the 95% confidence interval from the model in Section 2.6 was +43 to +167 gC m^−2^ (GLS mean +105.0 gC m^−2^). All five GCMs projected an increase (+84 to +133 gC m^−2^). However, the GCMs explained the smallest share of the variance (4.4%), compared to 41% for sites and 35% for species. The net change was negative in 11 of the 135 cells, compared to two with the seasonal reset. Konya and Eskişehir accounted for 64% of the decrease. The net change fell from +144.8 to +87.3 gC m^−2^ in Konya and from +111.5 to +51.0 gC m^−2^ in Eskişehir. At Antalya and Rize, the change was about 2 gC m^−2^ or less. In the 2020s and 2070s, the season started at relative soil moisture of 0.52 and 0.46, respectively, at Konya and 0.47 and 0.42, respectively, at Eskişehir. With the seasonal reset, the amount of water used by vegetation at these two sites (leaf transpiration multiplied by leaf area index [LAI]) was 76–96% of annual precipitation in the two time periods, compared with 69–76% with year-round soil water. The largest decrease was among *Q. coccifera* and *Q. cerris*, which fell from +153.2 and +68.9 to +102.1 and +29.0 gC m^−2^, respectively. The number of days above *T*_crit_ remained unchanged (12.2 per season in both cases in the 2070s).

Soil water strongly limits the modeled assimilation. With soil–plant hydraulics switched off, *A*_season_ was 832 and 1048 gC m^−2^ in the two windows, so Section 2.2 runs reached 54% and 55% of these values. With year-round soil water, the result therefore depends on how much water the stand receives and uses, and we changed one assumption at a time in this configuration (GFDL-ESM4, five-year windows; Figure 7b, Table A3). The reference net change was +132.4 gC m^−2^, compared with +156.2 gC m^−2^ for the same cells and years with the seasonal reset. The largest effect came from stand water use. The model withdraws from the soil the transpiration of a sunlit leaf multiplied by the full LAI, an upper bound on this water use. When only the sunlit leaf area (Section 4.7) withdrew soil water, which is a lower bound, the net change was +198.8 gC m^−2^. The value obtained with the seasonal reset lies between these two bounds. With 30% and 60% more precipitation, the net change was +152.1 and +164.3 gC m^−2^; with soil evaporation halved and doubled, it was +144.9 and +108.2 gC m^−2^. The two alternative soil textures in Section 2.8 (loam and loamy sand) shifted the net change by −5.1 and +1.9 gC m^−2^, less than half as much as with the seasonal reset, with the same signs. Five further variants each shifted it by 0.2 gC m^−2^ or less: solving the leaf energy balance without the linearization of Section 4.3, stepping the soil water balance hourly, starting the spin-up from a relative soil moisture of 0.45 or 0.75, and correcting the scale of the vulnerability curve (Section 4.2). The simplifications of the implementation described in Appendix D are tested in Section 2.12 (the last four rows of Table A3). The mean net change was positive under every assumption (+108 to +199 gC m^−2^), but its magnitude and the species ranking depend on the soil water balance. For example, the net change for *Q. coccifera* was +134.2 gC m^−2^ with the whole LAI and +304.2 gC m^−2^ with the sunlit leaf area (fourth and first among the eight species for these cells).

In the attribution of Section 2.2, the channel that is held at its baseline is taken from the paired baseline year (Section 4.6). Each of the three weather terms (warming, precipitation, and radiation) therefore combines the mean change in that channel with the difference between the weather of two particular years. As a check, we kept the weather of 2073–2077 as one sequence and removed from it the monthly mean change in one weather channel, or in all three, between 2020–2030 and 2070–2080 (weather in sequence; Section 4.7; five GCMs, seasonal reset; Figure 7c, Table A4). For these five years, the net change was +131.3 gC m^−2^. The warming term was −63.8 gC m^−2^ with the channel held at the paired baseline year and −59.4 gC m^−2^ with the weather in sequence. The precipitation term was −40.1 and −29.7 gC m^−2^, and the radiation term was −3.1 and −1.8 gC m^−2^. The CO_2_ term (+219.4 gC m^−2^) comes from the same runs in both cases. The residual of Equation (1) fell from +19.0 to +2.8 gC m^−2^. With the weather in sequence, this residual has two parts. The first is the non-additive part of the four mean changes (in CO_2_ and in the three weather channels), +6.3 gC m^−2^: the change caused by the four together minus the sum of the four terms. The second is the weather sequence term, −3.5 gC m^−2^ (−25.0 to +14.6 gC m^−2^ among the GCMs): *A*_season_ of 2073–2077 with all four mean changes removed minus that of 2023–2027. The non-additive part is thus about one-third of the residual obtained with the paired baseline year (+6.3 of +19.0 gC m^−2^; 12–54% in the individual GCMs), and the other two-thirds result from pairing the weather of different years. In the ensemble mean, both methods produced the same signs and order for the four terms. This test was run for SSP5-8.5 only, so it does not show how these shares change with the size of the perturbation.

Four further results concern the number of days above *T*_crit_ (Figure 7d, Table A5). First, it hardly depends on soil water. With hydraulics switched off, the number in the 2070s was 11.5 days per season instead of 12.2, and a leaf kept at the stomatal model’s residual conductance (0.01 mol m^−2^ s^−1^; Section 4.1) under the same forcing gave 12.2. With the weather in sequence, removing the mean change in precipitation left the number for 2073–2077 at 12.0, whereas removing the mean change in temperature lowered it to 1.6 (Table A4). Second, it depends on the threshold: a threshold 1 °C higher or lower gave 8.1 or 17.5 days. Third, it depends on the stomata. On the days above *T*_crit_, the modeled conductance was at or near its residual value, and net assimilation was negative on average (−1.0 gC m^−2^ per day in the 2070s). A leaf that kept a conductance of 0.05 or 0.10 mol m^−2^ s^−1^ would exceed *T*_crit_ on 6.0 or 2.2 days. The air temperature itself exceeded *T*_crit_ on only 0.5 days per season in the 2070s, so, on most of these days, the threshold was exceeded because the leaf was warmer than the air. Fourth, it depends on the air temperature assigned to the stands. The forcing is that of the grid cell and was not adjusted to the stands’ elevation. With the air 3 °C cooler at the same relative humidity, the number in 2073–2077 fell from 7.4 to 1.6 days, and with the air 6 °C cooler it fell to 0.2 days (GFDL-ESM4, Table A3). The net change rose from +132.4 to +137.5 and +140.4 gC m^−2^. At near-surface lapse rates of about 5–7 °C km^−1^, as reported for Türkiye and a southern Mediterranean region [29,30], these offsets correspond to about 400–600 m and 900–1200 m of elevation. For comparison, the mean air temperature of the conifer growing season rose by 2.9–3.5 °C between the two windows at the six sites (five-GCM means; Table A14). The effect of cooler air differed between the species groups. With the air 3 °C cooler, the number in 2073–2077 fell from 18.3 to 4.3 days per season for the broadleaved species and from 0.9 to 0 for the conifers, and with the air 6 °C cooler to 0.6 and 0 days; in 2023–2027, it was 0.1 days or fewer in both groups. At the six sites, the 2073–2077 number fell from 1.2–14.9 days to 0–3.3 days with 3 °C and to 0–1.1 days with 6 °C cooler air. The net change kept its sign at every site; it rose at five sites and fell only at Rize (from +220.7 to +200.8 and +181.0 gC m^−2^). Stand elevations could not be assigned, because each species–site combination represents the species’ occurrence in the province rather than a located stand, and these species occur from near sea level to about 2000–2200 m (Section 4.4). An elevation correction would therefore lower the counts most for montane stands and could remove the exceedances of the conifers, but not the increase in the broadleaved species. In every variant of Table A3 and Table A5, the number of days increased from the 2020s to the 2070s, and in every row of Table A5 it was larger for the broadleaved species than for the conifers.

### 2.10. Further Tests with Year-Round Soil Water

Five further analyses used year-round soil water (Section 4.7; Table A6, Table A7, Table A8, Table A9 and Table A10). In the first, the attribution was repeated as a full factorial design: for 2073–2077, with the five GCMs, all 16 combinations of the four channels with and without their mean change were run, with the weather of 2073–2077 kept in sequence (Section 2.9). The contribution of a channel can then be measured when it is changed first, with the other three at their baseline (first-in); when it is changed last, as in Section 2.2 (last-in); or as its Shapley value, the average over all orders in which the four channels can be changed. The Shapley values were +218.3 ± 5.2 gC m^−2^ for CO_2_, −63.2 ± 2.5 gC m^−2^ for warming, −41.4 ± 3.0 gC m^−2^ for precipitation, and −1.9 ± 0.2 gC m^−2^ for radiation (Table A6). They add up to the change caused by the four mean changes together (+111.9 gC m^−2^), which with the weather sequence term (−2.8 gC m^−2^) gives the net change of +109.1 gC m^−2^. The first-in and last-in contributions bracketed the Shapley values (CO_2_, +222.5 and +212.8 gC m^−2^; warming, −60.5 and −67.1; precipitation, −36.2 and −47.8; radiation, −1.8 and −2.1), and the largest two-way interaction was −5.5 gC m^−2^ (CO_2_ × precipitation). In the ensemble mean, all three measures gave the signs and order of the channels found in Section 2.2; in MPI-ESM1-2-HR, the precipitation contribution exceeded the warming contribution with all three. The approximate crossed random-effects intervals of the Shapley values (Section 4.9) retained their signs (CO_2_, +132 to +301 gC m^−2^; warming, −85 to −38; precipitation, −69 to −17; radiation, −4.0 to −0.2). With year-round soil water, the precipitation contribution included the effect of drier winters and was largest at Eskişehir (−73.5 gC m^−2^) and Konya (−59.6 gC m^−2^). These contributions remain conditional on the model: they describe how the modeled leaf responds to each forcing change in combination with the others, not separable physical effects.

In the second analysis, the last-in CO_2_ term (+212.8 ± 5.6 gC m^−2^; 2073–2077) was split by recomputing the leaf of the run without the CO_2_ change with the scenario C_a_ (Section 4.7; Table A7). The direct effect on assimilation at unchanged water status was +148.4 gC m^−2^ (70%); the adjustment of water status within the day contributed −3.5 gC m^−2^ (−2%); and soil water saved through lower stomatal conductance and carried forward to later days contributed +68.0 gC m^−2^ (32%). The carried-forward part was largest at Konya (+79.1 gC m^−2^) and smallest at Rize (+47.1 gC m^−2^), and it accounted for 30% of the CO_2_ term of the conifers and 36% of that of the broadleaved species. With the photorespiratory CO_2_ compensation point (Γ*) set to zero, the direct effect was +120.4 gC m^−2^, so 19% of the direct effect came from the reduced release of photorespiratory CO_2_.

In the third analysis, one trait at a time was changed for all species (GFDL-ESM4, five-year windows; Table A8). Relative to the reference net change of +132.4 gC m^−2^, LAI had the largest effect (×0.7, +34.9 gC m^−2^; ×1.3, −23.7), because a smaller leaf area withdraws less soil water, followed by carboxylation capacity (×0.7, −23.0; ×1.3, +16.2) and rooting depth (×0.5, −21.7; ×1.5, +15.7). Stomatal slopes g_1_ of 3 and 5 kPa^0.5^ instead of 4 changed the net change by +10.7 and −9.2 gC m^−2^, and shifting the turgor loss point by ±0.5 MPa changed it by less than 2.5 gC m^−2^. The net change remained positive in every variant (+108.6 to +167.3 gC m^−2^), at most one of the 27 cells declined, and the number of days above *T*_crit_ in 2073–2077 stayed between 7.27 and 7.40.

In the fourth analysis, SSP1-2.6 was run with the five GCMs (11-year windows; Table A9). *A*_season_ rose from 409.8 to 443.5 gC m^−2^ (+33.6 ± 3.1 gC m^−2^); all five GCM means were positive (+6.3 to +65.8 gC m^−2^), but 25 of the 135 cells declined. The approximate 95% interval from the five GCM means was +1 to +67 gC m^−2^, wider than the crossed random-effects interval of +11 to +60 gC m^−2^ (Section 4.9). The two scenarios started from different realizations of 2020–2030, whose *A*_season_ differed by 21.6 gC m^−2^ (−6.0 to +35.0 gC m^−2^ among the GCMs). We therefore compared the scenarios on their 2070–2080 levels, which is equivalent to measuring both changes from a common baseline: SSP5-8.5 exceeded SSP1-2.6 by 94.5 gC m^−2^, with a crossed random-effects interval of +34 to +148 gC m^−2^, and by 67.5 to 121.0 gC m^−2^ in the individual GCMs. Measured from each scenario’s own baseline, the difference was 72.9 gC m^−2^, so the own baselines absorbed 23% of the separation (17% for GFDL-ESM4 with the seasonal reset; Section 2.8). Because every GCM showed the same sign and the scenario difference was more than four times the standard deviation of the GCM means (20.8 gC m^−2^), the scenario effect is distinguishable from the variation among these five GCMs, although its magnitude is conditional on them. Days above *T*_crit_ rose from 1.8 to 2.8 per season under SSP1-2.6.

In the fifth analysis, the days above *T*_crit_ were recomputed for thresholds other than the assigned ones (Section 4.3), from the counts made at 1–3 °C below and above them (year-round soil water, five GCMs, 11-year windows; Table A10). With the deciduous value of 44 °C as a common threshold for all species, the ensemble number rose from 1.8 to 16.9 days per season between the two windows, compared with 1.5 to 12.2 with the assigned thresholds; in the 2070s, the conifers had 8.2 days instead of 2.4 and *Quercus coccifera* 30.4 instead of 20.8, and the deciduous species were unchanged at 32.3. With common thresholds of 43–47 °C, the ensemble number in 2070–2080 ranged from 24.2 to 4.0 days. For every common threshold and for each group shifted separately by 1–3 °C, the number increased from the 2020s to the 2070s in every group, and the broadleaved species exceeded the threshold more often than the conifers: by 3.2- to 8.1-fold with a common threshold, against 12.1-fold with the assigned thresholds. Under SSP1-2.6, the common threshold of 44 °C gave 2.2 and 3.6 days in the two windows.

### 2.11. Leaf-Temperature Distributions and the Limiting Process

**Figure 8:**
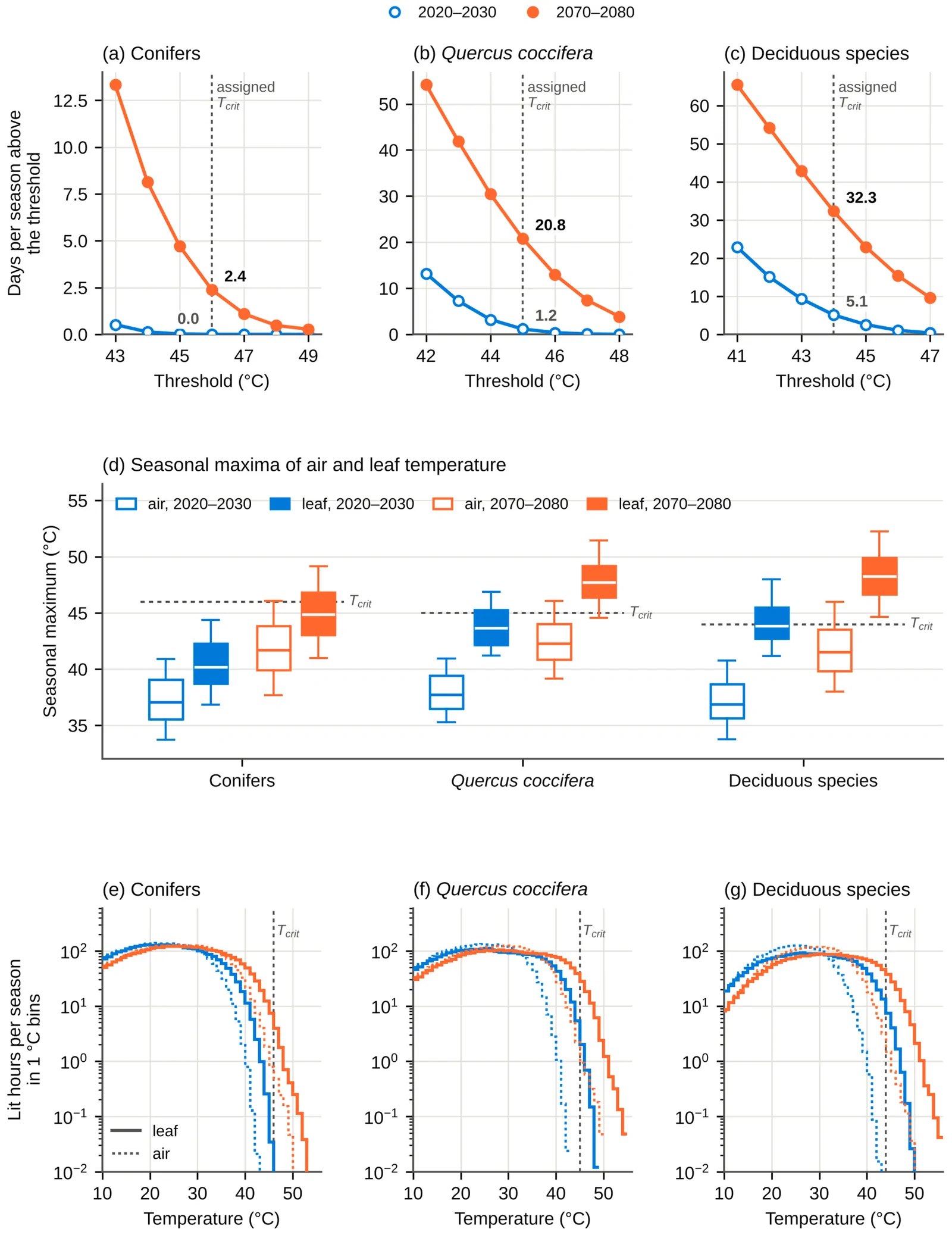
Leaf temperatures in the two windows under SSP5-8.5 (year-round soil water, five GCMs, 11-year windows, 135 cells). (**a**–**c**) The number of days per season on which the peak leaf temperature reached a given threshold, from 3 °C below to 3 °C above the assigned *T*_crit_ (dotted line), for (**a**) the conifers, (**b**) *Quercus coccifera*, and (**c**) the deciduous species; the labels give the numbers at the assigned *T*_crit_. (**d**) The seasonal maxima of air temperature (open boxes) and leaf temperature (filled boxes); the boxes show the interquartile range and the median, and the whiskers the 5th and 95th percentiles of all cell-years. (**e**–**g**) The hourly leaf temperature (solid lines) and air temperature (dotted lines) over the lit hours of the season, in hours per season in 1 °C bins (logarithmic scale; hours below 10 °C are not shown), from the runs with hourly output (Section 4.7). In (**d**–**g**), the dashed lines mark the assigned *T*_crit_.

The increase in the number of days above *T*_crit_ reflects a shift in the whole upper tail of the leaf-temperature distribution (Figure 8; year-round soil water, five GCMs, 11-year windows). The median seasonal maximum of leaf temperature rose from 40.2 to 44.9 °C in the conifers, from 43.7 to 47.7 °C in *Quercus coccifera*, and from 43.9 to 48.3 °C in the deciduous species, and that of air temperature rose by 4.6 °C in each group, more than the mean warming of the growing season (2.9–3.5 °C for the conifer season; Table A14). The seasonal maximum of the leaf–air temperature difference hardly changed (medians of 3.8 and 3.7 °C in the conifers, 6.9 and 6.6 °C in *Q. coccifera*, and 8.7 and 8.4 °C in the deciduous species). Because the thresholds lie in the steep upper tail of the distribution, this shift multiplied the number of days (Figure 8a–c): with their threshold of 44 °C, the deciduous species had 5.1 days per season in 2020–2030 and 32.3 in 2070–2080. The share of seasons in which the leaf reached its assigned threshold at least once rose from 0.2% to 34% in the conifers, from 29% to 93% in *Q. coccifera*, and from 48% to 97% in the deciduous species.

The daily maxima of leaf temperature (Section 4.7) show how much the number depends on two properties of the thresholds. First, *T*_crit_ varies among leaves: the source values of the deciduous threshold were 43.97 ± 2.38 and 44.25 ± 2.87 °C (means ± standard deviations of the individual measurements) [12]. With the threshold of each leaf treated as normally distributed with these standard deviations (Section 4.7), the expected number of days per season was 3.0–3.8 in 2020–2030 and 15.2–16.5 in 2070–2080, instead of 1.5 and 12.2 with fixed thresholds; with all thresholds one standard deviation lower or higher, it was 26.5–30.3 or 3.3–4.3 days in 2070–2080. Second, thresholds acclimate to growth temperature, by about 0.2–0.4 °C per °C (Section 4.3). With the thresholds of each species, site, and GCM raised by 0.2 or 0.4 °C per °C of the warming of its growing season (3.3 °C on average; 1.7–4.9 °C), the number in 2070–2080 was 9.0 or 6.5 days per season. In every case, the number in 2070–2080 was at least four times that in 2020–2030.

The leaf model explains why the number hardly depends on soil water (Section 2.9). For a leaf in full light without water stress, net assimilation falls to zero at a leaf temperature of 43.3 °C at 431.8 μmol mol^−1^ and 45.7 °C at 801.7 μmol mol^−1^ (C_i_ = C_a_), for every species, because day respiration is a fixed fraction of *V*_cmax25_ and *V*_cmax_ declines above its optimum of 31.7 °C (Appendix D). Above this temperature, the stomatal model returns its residual conductance (Section 4.1), transpirational cooling stops, and the leaf warms to the temperatures of Figure 8d; on the days above *T*_crit_, the conductance at the hottest step was 0.010 mol m^−2^ s^−1^ in both windows. At fixed conditions without water stress (Table A11), the sunlit leaf was limited by electron transport at 20–30 °C at both CO_2_ concentrations, because the ATP/NADPH budget spends 28–30% more electrons per CO_2_ than the FvCB expression, so that *J*_max25_ = 1.6 *V*_cmax25_ corresponds to an FvCB-equivalent ratio of about 1.24 (Appendix D). The CO_2_ stimulation between 431.8 and 801.7 μmol mol^−1^ at a constant C_i_/C_a_ was therefore the electron-transport-limited one, +18% at 20 °C and +29% at 30 °C, and it was the same for a leaf in one-sixth of the light; at 35 °C, above the *V*_cmax_ optimum, the sunlit leaf was Rubisco-limited and the stimulation was +102%. The photorespiratory CO_2_ release per carboxylation fell from 0.11–0.22 to 0.06–0.12, and the photons used per CO_2_ fixed fell from 12.6–17.4 to 10.8–13.0. Compared with the FvCB coefficients, the budget increased the electron-transport-limited CO_2_ stimulation by 1.0–1.9 percentage points.

The hourly leaf temperatures of the same runs show how long the thresholds were exceeded (Section 4.7; Figure 8e–g, Table A12). The hours per season at or above *T*_crit_ rose from 3.9 to 41.4 in the ensemble mean (of 2935 lit hours), from 0.0 to 7.2 in the conifers, from 2.8 to 68.0 in *Q. coccifera*, and from 13.9 to 113.1 in the deciduous species. On a day above *T*_crit_, the leaf stayed above it for 2.7 h on average in 2020–2030 and 3.4 h in 2070–2080, and for at most 6 and 8 h; the number of days with at least 4 h above *T*_crit_ rose from 0.5 to 6.9 per season (from 1.8 to 19.4 in the deciduous species), and the degree-hours above *T*_crit_ from 4.5 to 80.2 °C h. The hours above a threshold came in pairs because the sub-daily forcing is symmetric about noon (Equation (A12)); durations are therefore resolved to 2 h, and the lag of the afternoon temperature maximum is not represented. Over all lit hours, the leaf temperature reached 40 °C on 54.7 h per season in 2020–2030 and 230.4 h in 2070–2080, and the air temperature on 0.9 and 49.0 h (Figure 8e–g). In the paired measurements of the source data, the temperature at which the photosystem II efficiency falls by 50% (*T*_50_) lay 6.50 ± 2.93 and 5.32 ± 1.80 °C above *T*_crit_ [12]; peak leaf temperatures that far above *T*_crit_ occurred on 0.3 and 0.7 days per season in 2070–2080 (1.0 and 2.5 in the deciduous species) and on fewer than 0.01 days in 2020–2030.

### 2.12. Simplifications of the Implementation

Appendix D describes three simplifications of the implementation: the PAR curve spans a fixed 12 h, the transpiration charged to the soil omits the boundary-layer conductance, and Rubisco constants estimated on an intercellular basis are applied to the chloroplast CO_2_. We removed each of them in the configuration of Table A3 (GFDL-ESM4, year-round soil water, five-year windows) and, with the five GCMs, all of them together with the corrected scale of the vulnerability curve and the shape of the measured curve of *Pinus nigra* (Section 4.7). With GFDL-ESM4, the net change of +132.4 gC m^−2^ became +138.7 gC m^−2^ with the PAR curve over the astronomical day, which raised *A*_season_ in 2073–2077 by 4.3%; +129.6 gC m^−2^ with the boundary-layer conductance in the transpiration, which lowered transpiration in 2073–2077 by 1.7% and raised *A*_season_ by 3.5%; and +136.1 gC m^−2^ with the chloroplast-basis constants (Table A3). With the five GCMs, all five changes together raised *A*_season_ by 10.3% in 2023–2027 and by 9.4% in 2073–2077, and the net change from +109.1 to +115.6 gC m^−2^ (+6.5 ± 1.0 gC m^−2^): by 10.5 gC m^−2^ in the conifers and 0.5 gC m^−2^ in the deciduous species, and by −2.1 gC m^−2^ in *Q. coccifera* (Table A13). The net change declined in 11 instead of 10 of the 135 cells, and the number of days above *T*_crit_ in 2073–2077 rose from 12.0 to 12.1. The shape of the measured curve of *P. nigra* alone (*c* = 4.40 instead of 2.21) raised its net change by 0.1 gC m^−2^ (five GCMs).

With the chloroplast-basis constants and the five GCMs, the last-in CO_2_ term of Section 2.10 was +215.9 instead of +212.8 gC m^−2^, the last-in warming term −65.5 instead of −67.1 gC m^−2^, and the net change +112.8 instead of +109.1 gC m^−2^ (Table A13). The photorespiratory CO_2_ release was 12% lower in 2023–2027 and 16% lower in 2073–2077 (46.2 instead of 55.2 gC m^−2^), and the number of days above *T*_crit_ in 2073–2077 was unchanged (12.0). With the seasonal reset, for 2073–2077 relative to 2023–2027 as in Table A4, the CO_2_ term was +223.3 instead of +219.4 gC m^−2^, the warming term −61.9 instead of −63.8 gC m^−2^, and the net change +136.0 instead of +131.3 gC m^−2^; the warming of the 2070s raised the photorespiratory CO_2_ release by 1.8 instead of 4.3 gC m^−2^.

## 3. Discussion

### 3.1. Leaf Carbon Gain Under Rising CO_2_ Warming and Drying

Across the sampled species–site combinations, all five GCMs projected a positive mean change in seasonal leaf assimilation under SSP5-8.5. With year-round soil water, the ensemble increase was 106.5 gC m^−2^, and the approximate crossed random-effects interval was +43 to +167 gC m^−2^ (Section 2.9). The seasonal-reset configuration gave +130.3 gC m^−2^, with an interval of +76 to +187, while the common historical baseline gave an interval of +86 to +225. These differences show that the estimated magnitude depends on the water-balance and baseline assumptions. Positive ensemble means do not imply gains in every cell: 11 of 135 cells declined with year-round soil water. The intervals are conditional on the specified model and omit uncertainty in assumed traits and canopy representation. Under SSP1-2.6, the five-GCM increase was smaller (+33.6 gC m^−2^), and 25 of the 135 cells declined (Section 2.10).

The CO_2_ contribution (+219 gC m^−2^) is the largest component in the attribution, and it comes from a model that does not include sink feedback or nutrient limitation, factors that would reduce it. It remained the largest contribution in every order in which the four channels can be changed (+212.8 to +223.0 gC m^−2^; Section 2.10), and about one-third of it came from soil water saved by lower stomatal conductance rather than from faster carboxylation. Tian et al. [31] found that hexose accumulation reduced the photosynthetic capacity of mature boreal silver birch; this feedback, observed in a different biome, is missing from our model. When we apply the down-regulation rate of carboxylation capacity derived from the FACE experiments, the ensemble gain decreases by 8% (also 8% in the case of GFDL-ESM4); when we double this down-regulation, the gain of GFDL-ESM4 decreases by 27%. The modest size of this penalty is partly due to the fact that a leaf containing less Rubisco also transpires less and so soil water is retained for a longer period, which offsets part of the lost capacity (Section 2.5). This test examines only one process that could reduce the effect of CO_2_. The sign of the mean change persisted under the tested acclimation rates, but it still depends on the absence of nutrient or sink limitation.

The processes that limit the CO_2_ gain in experiments act on parameters that the model holds fixed. Photosynthetic acclimation lowered *V*_cmax_ by 13% and Rubisco content by 20% on average in FACE experiments, and more at low nitrogen supply [13]; this is attributed to the accumulation of carbohydrates, possibly sensed through hexokinase, which lowers the synthesis of the Rubisco small subunit [32]. The same imbalance between source and sink can appear as a limitation by triose phosphate use, which becomes more frequent at high CO_2_ and less frequent with warming and is omitted from many global models [33], as it is from ours (Appendix D). Leaf nitrogen per unit mass of woody plants was 16% lower at about twice the ambient CO_2_ [34], through dilution by additional carbohydrates and lower transpiration-driven uptake of nitrogen [35], and specific leaf area was 6% lower in FACE experiments, so leaf mass per area was higher [13]. Stomatal density in herbarium leaves of eight temperate tree species fell by 40% over two centuries of rising CO_2_ [36], although it changed little in FACE experiments [32]; the model represents the stomatal response to CO_2_ only through the optimal conductance of Equation (A9). The down-regulation test of Section 2.5 therefore stands for several processes that act in the same direction, a lower photosynthetic capacity per unit leaf area at high CO_2_.

Temperature acts on the same terms. Γ*, *K*_c_, and *K*_o_ increase with temperature (Table A15) [37]; Γ* rises mainly because the specificity of Rubisco for CO_2_ relative to O_2_ declines [38], so that at current CO_2_ the ratio of carboxylation to oxygenation falls from about 2.2 at 25 °C to 1.4 at 35 °C [32], and elevated CO_2_ shifts the temperature optimum of photosynthesis upward [39]. Part of the warming term is therefore a photorespiratory penalty. In the attribution of Section 2.2, the temperatures of the 2070s raised the photorespiratory CO_2_ release in 2070–2080 from 55.0 to 59.4 gC m^−2^, from 7.9% to 9.3% of gross assimilation, so this extra release accounts for 4.4 gC m^−2^, about 7%, of the warming term of −66.7 gC m^−2^. With C_a_ at its 2025 value, the same warming raised the photorespiratory share from 14.6% to 17.5% (factorial runs, Section 2.10), so the penalty is larger at present CO_2_. The rest of the warming term is lower gross assimilation, which also contains the effect of the higher Γ* and *K*_c_ on carboxylation, the higher vapor pressure deficit, faster respiration, and the decline of *V*_cmax_ above its optimum. The decline of Rubisco activation at moderately high temperatures, which is linked to the thermal lability of Rubisco activase [39], is represented only implicitly, through the peaked temperature function of *V*_cmax_ (Equation (A8)). The kinetic constants, moreover, were estimated on an intercellular CO_2_ basis, that is, for an infinite mesophyll conductance [37], but are applied here to the chloroplast CO_2_ of a leaf with a finite conductance; with the chloroplast-basis constants [40], the CO_2_ stimulation of the leaf of Table A11 was +20% instead of +23% at 25 °C and +23% instead of +29% at 30 °C (Appendix D); over the season, however, these constants raised the CO_2_ term by 3.1 gC m^−2^ with year-round soil water and by 4.0 gC m^−2^ with the seasonal reset, and the warming of the 2070s raised the photorespiratory CO_2_ release by 1.8 instead of 4.3 gC m^−2^ (2073–2077; Section 2.12).

How much of the leaf-level gain would survive at the stand scale cannot be estimated with a single sunlit leaf, but forest FACE experiments show that it shrinks at each step from leaf to wood. Shaded leaves gained less: in a sweetgum canopy, the daytime carbon gain rose by 98% in sun leaves and by 41% in shade leaves at elevated CO_2_ [41], and in a mature oak canopy the enhancement fell from 30% at high light to 7% at low light [42]. In our model, a leaf in one-sixth of the light had the same relative stimulation as the sunlit leaf at 20–30 °C, because both were limited by electron transport (Section 2.11), so canopy shading would mainly reduce the absolute gain; at 25 °C the stimulation (+23% for an increase of 370 μmol mol^−1^; Table A11) was smaller than the 47% increase in light-saturated photosynthesis of trees in FACE experiments at about 200 μmol mol^−1^ above ambient [13]. Beyond the leaf, the net primary productivity (NPP) of four young forest stands increased by a median of 23% at about 550 μmol mol^−1^, about half as much, and 11–93% of the additional NPP went to wood [43]. Where nitrogen limited growth, the NPP enhancement declined from 24% to 9% [20]; on a phosphorus-poor soil, leaf photosynthesis increased by 19% [44] and gross primary productivity by 12% without an increase in ecosystem carbon storage [21]; and in one stand none of the additional NPP remained in biomass at the end of the experiment [45]. Globally, nitrogen and phosphorus are estimated to limit the CO_2_ fertilization of plant biomass to 12 ± 3% for the CO_2_ concentrations expected by 2100 [46]. The modeled gain is therefore an upper bound for stand growth rather than an estimate of it.

Our findings differ from those of Sperry et al. [5], who found that, without acclimation, rising C_a_ did not prevent chronic hydraulic stress in 45% of their projections for US forests and that acclimation of leaf area, photosynthetic capacity, and stand density could not counteract warming-induced declines in stand net primary productivity in the warmer projections. Three differences could account for this, and our study cannot tell which applies. First, their model deals with stands and incorporates canopy processes and carbon allocation, while ours focuses on a single leaf. Second, their study includes a whole continent with many biomes, whereas ours focuses on a gradient from the Mediterranean to the Black Sea within a single country, where annual precipitation is projected to decrease by 2–16% at the six sites (Table A14). The third point is that the sites where compensation failed in their study also experienced greater warming. One possible explanation—something a leaf model cannot test—is that stand-level processes, such as carbon allocation and respiration, reduce CO_2_ gains at the leaf level.

### 3.2. Ensemble Heterogeneity and Design Sensitivity

The variance partition provides information about the sampled ensemble rather than a universal ranking of uncertainty sources. Species and site effects accounted for approximately 50% and 31% of the fitted variance, whereas GCM effects accounted for 3.1%. The species and site components include biological and geographical heterogeneity as well as differences introduced by assumed traits and the sampling design. They are relevant to estimation of an ensemble mean but should not be interpreted as uncertainty in each individual forest. Shared bias adjustment and prescribed CO_2_ forcing may also limit the apparent spread among GCMs. Consequently, reporting GCM spread alone would omit substantial variation represented in this design, while even the crossed random-effects interval omits structural and parameter uncertainty. The intervals in Section 2.6 and Section 2.10 are therefore conditional on the sampled species, sites, and GCMs, on the shared forcing protocol, and on the model structure and trait values; they are not prediction intervals for other forests, other climate models, or other model structures.

Two design decisions had a greater effect than we anticipated. The first of these concerned the sampling unit. In this experiment, one-year time slices were noisy and potentially misleading, as they caused the various GCMs to appear to disagree with one another, including showing a reversal of sign, a discrepancy that vanished when 11-year windows were used. The reason for this is clear. In the one-year simulations, the precipitation amount in the 2075 slice year reduced assimilation compared to that in the 2025 slice year by 149, 94, 113 and 56 gC m^−2^ for GFDL-ESM4, IPSL-CM6A-LR, MRI-ESM2-0 and UKESM1-0-LL respectively, but by only 3 gC m^−2^ for MPI-ESM1-2-HR, because the two slice years of this model happened to have similar precipitation anomalies with respect to their decades. Studies that base their GCM comparisons on single years of Mediterranean precipitation might end up attributing sampling error to structural uncertainty.

The second decision concerns the choice of baseline. Although it may seem harmless to calculate the change for each scenario relative to its own early period, in ISIMIP3b both pathways diverge from the same historical simulation in 2015, so their early periods are two different versions that differ mainly because of weather. Using the last decade common to both scenarios (2005–2014) lowered the ensemble change per decade by 10% (from +26.1 gC m^−2^ with each scenario’s own baseline to +23.4 gC m^−2^ with the common one), and for GFDL-ESM4 it increased the difference between SSP1-2.6 and SSP5-8.5 from 85.5 to 102.7 gC m^−2^ (a 1.2-fold increase). Thus, the scenario-specific baselines have absorbed about 17% of the separation that would have been obtained using the common baseline. With the five GCMs and year-round soil water, they absorbed 23% (Section 2.10). We present both types of baseline and advise using the common one when the aim is to compare different pathways rather than describe a single pathway.

We report the residual separately rather than allocate it to individual forcing channels. For SSP5-8.5, it was +19 gC m^−2^ (15% of the total change), and for GFDL-ESM4 it fell from +14.4 to +3.4 gC m^−2^ when going from SSP5-8.5 to SSP1-2.6. A dependence on the magnitude of the perturbation is to be expected in a leave-one-out decomposition of a coupled system, but the weather of the paired years also contributes to the residual. The full factorial design (Section 2.10) shows how large this non-additive part is when the weather is kept in sequence: +16.0 gC m^−2^ relative to the last-in contributions and −12.1 gC m^−2^ relative to the first-in contributions, with two-way interactions of at most 5.5 gC m^−2^. The Shapley values allocate it among the channels, but such an allocation is a convention rather than a physical interaction, and attribution methods that enforce additivity without reporting it conceal the non-additive part of the change. Any studies that give exact percentage splits among the different drivers, such as our earlier estimate based on expert elicitation [11], implicitly assume that this term is zero. For 2073–2077, the non-additive part of the four mean changes was +6.3 gC m^−2^ when the weather was kept in sequence, compared with a residual of +19.0 gC m^−2^ (Section 2.9). Thus, about two-thirds of the residual reflects pairing weather from different years.

### 3.3. Leaf Carbon Gain and Thermal Threshold Exceedance

The conclusions from the carbon projection and the thermal projection differ: although leaves take up more carbon, they also exceed their thermal thresholds more frequently, and the species that achieve the greatest carbon gains are not those with the most days above *T*_crit_. This discrepancy should be examined using other models and with observational data. A forest in which leaves fix more carbon per unit area but exceed their photosystem II threshold on eight times as many days cannot be properly described by either of these figures alone; to quantify both, a model needs a leaf energy balance, plant hydraulics, and an assigned thermal threshold at the same time. MEDFATE [6] includes the first two elements but not an explicit thermal threshold, and the species distribution models [7,8,9,10] include none of them. In our model, however, the increase in days above T_crit_ is an effect of warming, not of the hydraulic–thermal sequence observed by Kullberg et al. [12]. With temperature and vapor pressure deficit held at their baseline values, the number in the 2070s (2070–2080) was 1.3 days per season instead of 12.2; holding precipitation at its baseline changed it by 0.02 days. Stomatal conductance follows assimilation in the model (Section 4.1), and net assimilation of a leaf in full light falls to zero at 43.3 °C at present and at 45.7 °C at the C_a_ of 2075 (Section 2.11), close to the assigned thresholds of 44–46 °C; the stomata are then at their residual conductance whether or not soil water is limiting, transpirational cooling stops, and the leaf–air temperature difference reaches its seasonal maximum of 3.7–8.4 °C (medians in 2070–2080; Section 2.11). The number of days rises steeply because the seasonal maximum of air temperature rose by 4.6 °C while this offset stayed nearly constant, so the upper tail of the leaf-temperature distribution moved across fixed thresholds (Figure 8). With soil–plant hydraulics switched off, the number in the 2070s was 11.5 days per season, whereas Aseason was 1.8–1.9 times as large (Section 2.9). The hydraulic part of the model therefore matters for the carbon result but hardly for the number of days. These exceedance counts do not establish cumulative thermal injury or its feedback on later carbon gain.

Real leaves need not follow this sequence. Stomata of well-watered loblolly pine and poplar opened between 30 and 40 °C while net photosynthesis became negative above about 40 °C, which cooled well-watered poplar leaves by up to 9 °C but drought-stressed ones by only about 1 °C [47]; across 207 warming studies, transpiration increased by 26% while net photosynthesis decreased by 10% and stomatal conductance did not change significantly, and drought constrained transpirational cooling [48]. In Mediterranean *Quercus ilex* during a heatwave, by contrast, stomatal conductance stayed near 0.01 mol m^−2^ s^−1^ while net assimilation was negative, and the canopy was on average 5.3 °C and up to 10 °C warmer than the air [49], close to the behavior of the model. The residual conductance of the model, 0.01 mol m^−2^ s^−1^, exceeds the minimum leaf conductances measured at 20 °C in European pine, spruce, beech, and oaks (0.8–2.7 mmol m^−2^ s^−1^), which increased two- to four-fold between 25 and 50 °C [50]; in tropical trees, cuticular conductance at 50 °C remained more than ten times lower than the peak leaf conductance [51], too low to cool a leaf appreciably. The numbers of days for a leaf that keeps a conductance of 0.05 or 0.10 mol m^−2^ s^−1^ (6.0 and 2.2 in the 2070s instead of 12.2; Section 2.9) thus bracket the effect of stomatal opening in heat in well-watered leaves, whereas under drought the number at residual conductance is the more relevant one. Exceeding *T*_crit_, moreover, marks the onset of photosystem II inactivation rather than tissue damage. Heat increases the fluidity of the thylakoid membranes, which impairs photosystem II [52], inactivates the oxygen-evolving complex, and inhibits the repair of photodamaged photosystem II, because reactive oxygen species suppress the synthesis of the D1 protein [53,54]. In saplings of three conifers, *T*_crit_ did not change with exposure duration, and photosynthesis recovered within four days from heating below *T*_50_, whereas *T*_50_ decreased with longer exposure [55]; in three Mediterranean oaks including *Q. coccifera*, *T*_50_ fell by 1.8–7.6 °C between 15 and 240 min of heating [56], and in two Australian species by 6.5 and 8.3 °C for each ten-fold increase in duration [57]. A daily count of peak exceedance is therefore an index of exposure to the onset of reversible photosystem II inactivation; whether damage accumulates depends on how long and how often the threshold is exceeded; in the model, a leaf that reached *T*_crit_ stayed above it for 2–8 h that day (3.4 h on average in 2070–2080), and its peak reached the level of *T*_50_ in the source data (*T*_crit_ + 5.3 to 6.5 °C) on 0.3–0.7 days per season (Section 2.11).

In this context, we should refer to the benchmark comparison (Section 2.7). At the ground-area scale, the carbon and water fluxes estimated by the model were at or above the upper end of the measured values for the same kinds of forest, and the modeled water-use efficiency was within the normal range, even though this merely constitutes a plausibility check and not an independent validation. The modeled leaf temperatures for the pines were plausible when compared with the measurements taken in a pine forest [28], but the broadleaved species, for which the majority of days above *T*_crit_ occurred, showed much larger leaf–air temperature differences, for which no corresponding measurements exist. The broadleaved species also have lower assigned *T*_crit_ values, some of which are assumed (Section 4.3); because direct comparisons do not support higher thresholds for conifers (in five temperate European tree species, the broadleaves were more heat tolerant than the conifers [58]), the conifers’ counts with the assigned threshold may be too low: with a common threshold of 44 °C, they rose from 2.4 to 8.2 days per season in the 2070s, while the broadleaved species still exceeded the threshold about four times as often (Section 2.10). Moreover, the energy balance parameters alone changed the number of days above *T*_crit_ by a factor of 5.7. We therefore present the thermal result in terms of direction rather than frequency: by the 2070s under SSP5-8.5, the modeled leaves exceed their thresholds on several times as many days as in the 2020s (at least 3.7-fold in every energy balance variant for GFDL-ESM4 and in every GCM). The carbon result is insensitive to the energy balance parameters (−4.0% to +3.8%), while the thermal result depends on them only in terms of magnitude. The frequency also depends on the air temperature assigned to the stands: with the air 3 °C cooler, the number of days in 2073–2077 was 1.6 instead of 7.4 for GFDL-ESM4 (Section 2.9). For the conifers, air 3 °C cooler, equivalent to about 400–600 m of elevation, removed the exceedances in both windows.

### 3.4. Limitations

The model’s most significant structural drawback is that it has no canopy. Instead, it simulates a single sunlit leaf, omitting self-shading, differences between sunlit and shaded canopy parts, and a canopy humidity profile. This probably accounts for the fact that the fluxes calculated by the model are at or above the upper end of the published ranges and that the ground-area values in Table 6 are arithmetic upper limits rather than actual simulation results; it also implies that the leaf temperatures obtained from the model correspond to the warmest, fully sunlit leaves in the canopy. With soil water withdrawn by the whole LAI or by the sunlit leaf area only, the GFDL-ESM4 net change was +132.4 or +198.8 gC m^−2^ (Section 2.9); a canopy with sunlit and shaded leaves would lie between these bounds, and so would its ground-area fluxes (Section 2.7). We tested three other assumptions directly (Section 2.8): separate baselines for the two scenarios, a fixed calendar growing season, and a single soil texture; the ensemble mean remained positive in the tested growing-season and soil variants. Other limitations still exist: only two of the 48 trait values were measured for the species in question, the SSP1-2.6 attribution used only one GCM, and, in the main runs, each season begins with the same relative soil moisture (Section 2.9 gives the result without this assumption). In addition, the stomatal model ties conductance to assimilation, so a leaf near *T*_crit_ is hardly cooled by transpiration even when water is available. Trees have been observed to keep transpiring through a heatwave while photosynthesis was close to zero [59], so the number of days above *T*_crit_ may be too high for such leaves. The stomatal model also uses one slope parameter for all species, g_1_ = 4.0 kPa^0.5^, which is typical of angiosperm trees; for evergreen needleleaf trees, a mean of 2.35 kPa^0.5^ has been fitted [60]. Conifers also reduce their stomatal conductance less at elevated CO_2_ than angiosperms, by about 2% against 6–8% per 100 μmol mol^−1^ [61], as also found in syntheses of field experiments [62,63]. The model does not represent this difference: the CO_2_ increase lowered the seasonal leaf transpiration of the conifers by 9.9% and that of the broadleaved species by 3.4%. The water saved, 30% of the conifers’ CO_2_ contribution (Section 2.10), is therefore probably overestimated for the conifers. With a lower g_1_, optimal stomatal theory reproduced the CO_2_ responses of stomatal conductance in gymnosperm as well as angiosperm trees [64]; in our model, g_1_ = 3 kPa^0.5^ raised the net change by 10.7 gC m^−2^ (Table A8), so a conifer-specific g_1_ would partly offset this bias. Moreover, the forcing is from the 0.5° grid cell nearest each site and was not adjusted to stand elevation. The stand water use is the transpiration of a sunlit leaf multiplied by the whole LAI, which is an upper bound. Section 2.9 shows how much the results depend on these two assumptions. Three further simplifications of the implementation, the fixed 12 h radiation curve, the omission of the boundary layer from the transpiration charged to the soil, and the use of intercellular-basis Rubisco constants with a finite mesophyll conductance, are described in Appendix D; with all three removed and the vulnerability curves corrected, the net change was 6.5 gC m^−2^ (6%) larger and the number of days above *T*_crit_ in 2073–2077 rose by 0.1 (Section 2.12). The most significant improvement to the model would be to include a canopy formulation that accounts for both lit and shaded areas, along with a within-canopy humidity profile. Thermal thresholds are exposure diagnostics; damage duration and recovery are not quantified by the reported day counts. The thresholds themselves are assigned by leaf habit rather than measured for these species, and the higher values for the sclerophyll and the conifers are not supported by direct comparisons (Section 4.3). Both the stomatal behavior in heat and the thresholds could be constrained by measurements at finer scales. Solving the leaf energy balance inside the stomatal optimization reproduces the opening of stomata at high temperature [65], and single-cell and spatial transcriptomics now map the stress responses of individual plant cell types [66]; applied to the guard cells and mesophyll of the modeled species under heat and drought, such data could show which cell type limits stomatal and photosystem II function first. The parameters themselves, however, require gas exchange, leaf temperature, and chlorophyll fluorescence measured on these species, including the duration of heating that leads to irreversible damage. Year-round simulations retain the fixed growing-season assumption and omit off-season transpiration, so they are not a complete annual vegetation water balance.

## 4. Materials and Methods

### 4.1. Leaf Model

The photosynthesis of leaves was calculated using the Farquhar–von Caemmerer–Berry (FvCB) model [67,68], implemented in a general two-compartment solver that simplifies to the FvCB equations for C_3_ leaves. The model is coupled to an explicit account of the supply and demand of ATP and NADPH, so that the energy requirements of both carbon fixation and photorespiration are calculated rather than assumed. For the budget, we used h = 4.67 H^+^ per ATP for a c_14_ ATP synthase [69], which makes cyclic electron flow necessary, and applied a spectral correction factor f = 0.15 to account for light not absorbed by the photosystems [68]. We determined the quantum yield as the exact ratio of gross assimilation to absorbed photons, as shown in the budget, rather than calculating it by finite differences. The non-photorespiratory limit of the budget is 9.005 photons per CO_2_, or 0.1110 mol CO_2_ mol^−1^ photons, against a value of 0.111 measured in red light by Evans [70]; no parameters were fitted. Stomatal conductance is based on the optimality model of Medlyn et al. [71]. In this model, stomatal conductance to water vapor is g_s_ = g_0_ + 1.6(1 + g_1_/√*D*)*A*/C_a_: a residual conductance g_0_ = 0.01 mol m^−2^ s^−1^ plus a term proportional to the net assimilation rate *A* divided by the atmospheric CO_2_ mole fraction C_a_, where *D* is the vapor pressure deficit (kPa) and g_1_ = 4.0 kPa^0.5^ for all species. CO_2_ therefore acts on g_s_ directly as well as through *A*: at a given *A* and *D*, a higher C_a_ lowers g_s_, and, apart from the residual conductance, the ratio of intercellular to atmospheric CO_2_ depends only on *D*. Because the ATP/NADPH budget spends 28–30% more electrons per CO_2_ fixed than the FvCB expression, the ratio *J*_max25_/*V*_cmax25_ = 1.6 used for seven species (1.73 for *Quercus coccifera*) corresponds to an FvCB-equivalent ratio of about 1.24 (1.34). The equations and parameter values of the leaf model and of the soil–plant hydraulics are given in Appendix D (Equations (A1)–(A16), Table A15 and Table A16).

### 4.2. Soil–Plant Hydraulics

Soil water was represented by a single layer extending to the assigned rooting depth, with sandy loam used at all sites in the reference configuration. A single layer was used because the rooting depths and root distributions of these stands are not known (all eight rooting depths are assumed; Appendix A); instead of resolving layers, we varied the size of the store: halving the rooting depth or increasing it by half changed the net change by −21.7 and +15.7 gC m^−2^ (GFDL-ESM4; Section 2.10). Soil–root and plant conductances act in series, and stomatal closure is imposed at the turgor loss point. Relative soil moisture is defined as the fraction of pore space occupied by water. In the seasonal-reset configuration, it starts at 0.60 and changes daily with precipitation, leakage, soil evaporation, and transpiration. Soil withdrawal is calculated as sunlit-leaf transpiration multiplied by the full LAI. Section 2.9 evaluates year-round soil water and withdrawal by sunlit leaf area only. Vulnerability was parameterized using stem P_50_; root vulnerability was not represented separately. Froux et al. [72] found roots to be 1.7–3.0 MPa more vulnerable than shoots in Mediterranean conifers. For *Pinus brutia*, the cited root P_50_ of −1.87 MPa is less negative than the assigned stomatal closure potential of −2.6 MPa. This comparison indicates that appreciable root conductivity loss could precede closure; 50% conductivity loss does not imply complete root failure or mortality. Omitting separate root vulnerability may therefore underestimate hydraulic limitation. It cannot be added simply by using root P_50_ in the single vulnerability curve: with root P_50_ values 1.7 MPa less negative than the stem values, the smallest offset found in Mediterranean conifers [72], the curve would remove 76% (*Picea orientalis*), 86% (*Pinus brutia*), and nearly all (*P. nigra*) of the plant conductance before stomatal closure at the turgor loss point, so that closure would no longer protect the hydraulic path. Representing root vulnerability requires a separate root compartment with its own regulation, which we did not attempt. In the reference implementation, the Weibull scale is |P_50_|, giving 37% remaining conductance at that potential rather than 50%. This is an implementation mismatch with the physiological definition of P_50_, not a redefinition of that trait. With the corrected scale, the net change differed by less than 0.2 gC m^−2^ in each of the 27 species–site cells (GFDL-ESM4, year-round soil water, five-year windows; Table A3), and seasonal assimilation by at most 0.4 gC m^−2^ in any season. The reason applies to every species: at the turgor loss point, where the stomata close, the reference curve retains 45–88% of the plant conductance and the corrected curve 57–91%, while in drying soil the steep decline of the soil–root conductance limits the supply first. The test was run with one GCM and was not repeated with the other four. The equations of the water balance and of the hydraulic path are given in Appendix D.

### 4.3. Leaf Energy Balance and Thermal Thresholds

We did not prescribe leaf temperature; instead, we calculated it using the linearized Penman equation from Campbell and Norman [73] to avoid solving a nested nonlinear equation during the stomatal iteration. Solving the energy balance without the linearization changed the number of days above *T*_crit_ in 2073–2077 from 7.38 to 7.17 and shifted the net change by 0.2 gC m^−2^ (GFDL-ESM4; Table A3). We determined boundary-layer conductance by leaf habit (3.0 for evergreen conifers, 1.6 for evergreen sclerophyll, and 1.2 mol m^−2^ s^−1^ for deciduous leaves), so needles are more strongly coupled thermally to the surrounding air than broader leaves. We fixed broadband shortwave absorptance at 0.50, below that for photosynthetically active radiation, since near-infrared absorptance is low. We assigned critical photosystem II temperatures (*T*_crit_) according to leaf habit. The value for deciduous leaves, 44.0 °C, is based on Kullberg et al. [12], who reported 44.25 ± 2.87 °C for *F. sylvatica* and 43.97 ± 2.38 °C for *Q. pubescens*, both of which are deciduous, and found no difference between their four treatments; the higher values of 45.0 °C for sclerophyll leaves and 46.0 °C for conifer leaves are assumptions. They were meant to reflect the thicker, longer-lived leaves of these groups, but the published evidence does not support a systematic difference. In direct comparisons, conifer needles were not more heat tolerant than broadleaves: the onset temperatures of the decline in photosystem II efficiency of six temperate conifers, including *Pinus nigra* (43.1 °C), averaged 40.3 °C [74], and the two conifers among 15 temperate tree species had lower thresholds of 50% loss than beech and oak [75]. For evergreen oaks the evidence is mixed: *Quercus coccifera* and *Q. ilex* were 1–6 °C more tolerant than the marcescent *Q. faginea* at one site [56], whereas their fluorescence-rise thresholds (51.4 and 53.1 °C) were lower than that of the deciduous *Q. petraea* (59.5 °C) measured at another site [76]. Thresholds also acclimate to growth temperature by about 0.2–0.4 °C per °C [77], and in heat-tolerance assays they depend on the duration of heating by several degrees [56]. We kept the assigned values so that the results remain comparable across sections, and we report the days above a common threshold for all species and above group-specific shifts of 1–3 °C (Section 2.10, Table A10). Because none of these thresholds was measured on the modeled species, the counts are exposure diagnostics rather than species-specific estimates. *T*_crit_ is the onset of the rise in basal chlorophyll fluorescence and thus of photosystem II inactivation, which is largely reversible, whereas irreversible damage begins near *T*_50_ [55]; in oak saplings including *Q. coccifera*, *T*_crit_ rose by about 4–5 °C when the growth temperature was raised from 15 to 35 °C [78]. A day above *T*_crit_ is defined as a day on which the peak modeled leaf temperature exceeds *T*_crit_. Sub-daily forcing uses 24 bin-averaged steps per day to resolve the daily temperature peak. Exceedance is a diagnostic of potential thermal exposure; the daily count alone does not represent exposure duration, irreversible tissue damage, or recovery.

### 4.4. Species and Sites

At six locations—namely the Mediterranean coast (Antalya), the Aegean and southern Marmara regions (Balıkesir), the Central Anatolian steppe and the area transitional to it (Konya and Eskişehir), the eastern Mediterranean hinterland (Kahramanmaraş) and the humid northeastern Black Sea coast (Rize)—simulations were carried out for eight species (see Figure 1). The forcing for each site is that of the 0.5° ISIMIP3b grid cell nearest to the site coordinates (Section 4.5): Antalya, 36.90° N, 30.70° E; Balıkesir, 39.65° N, 27.89° E; Konya, 37.87° N, 32.48° E; Eskişehir, 39.77° N, 30.52° E; Kahramanmaraş, 37.58° N, 36.92° E; and Rize, 41.02° N, 40.52° E. The forcing was not adjusted to the elevation of the stands (Section 2.9). Stand elevations could not be assigned, because each species–site combination represents the species’ natural occurrence in the province rather than a located stand; Section 2.9 reports the effect of air 3 and 6 °C cooler than that of the grid cell, by species group and site. The results are given only for species–site combinations within the documented natural range (in-range combinations; Table 8), and only these combinations were simulated.

*Quercus coccifera* represents the evergreen sclerophyll group. Akkemik et al. [79] describe *Q. ilex* in Türkiye as a fragmented coastal species that is often confused with *Q. coccifera*. We therefore used the documented distribution of *Q. coccifera*, a dominant maquis species [80], for the modeled species–site combinations. Its physiological parameters were nevertheless compiled from *Q. ilex* and treated as surrogate values (Appendix A). This substitution limits species-specific interpretation. Although *Q. coccifera* may be more drought tolerant, the surrogate parameter set does not establish that all projected stress effects are upper bounds.

**Table 8:** The occurrence of the study species at the six sites. ✓ indicates a natural occurrence that has been documented; P means it is found only in plantations (and has therefore been excluded); — shows that it is absent. The sources are listed in the footnote.

Species	Antalya	Balıkesir	Konya	Eskişehir	Kahramanmaraş	Rize
*Pinus brutia* ^a^	✓	✓	P	P	✓	—
*Pinus nigra* subsp. *pallasiana* ^b^	✓	✓	✓	✓	✓	—
*Cedrus libani* ^c^	✓	P	✓	P	✓	—
*Juniperus excelsa* ^d^	✓	✓	✓	✓	✓	—
*Quercus coccifera* ^e^	✓	✓	—	—	✓	—
*Quercus cerris* ^f^	✓	✓	✓	✓	✓	—
*Fagus orientalis* ^g^	—	✓	—	—	—	✓
*Picea orientalis* ^h^	—	—	—	—	—	✓

^a^ [81,82]; since the natural range does not extend across the Taurus watershed, afforestation occurs in the interior areas. ^b^ [83,84,85]. ^c^ [86,87]; ‘Eskişehir’ in the list of certified seed stands is an administrative forest directorate; the stand itself is located at Sultandağı in Afyonkarahisar. ^d^ [88,89]. ^e^ [79,80]. ^f^ [90,91]; according to the EUFORGEN guideline, the northern Anatolian limit is Samsun, so Rize is excluded. ^g^ [92,93,94]; the microrefugial population noted at Akseki [95] is a relict area of approximately 0.5 ha at an altitude of 1750–1800 m and is too small to be treated as an occurrence at this site. ^h^ [96,97]. In the Fırnız basin in Kahramanmaraş, *P. brutia*, *P. nigra*, *C. libani*, *J. excelsa* and *Q. cerris* are recorded [98], and there are certified *C. libani* seed stands in the province [87]; *Q. coccifera* is the most common of the five native oak taxa around the city [99]; neither [98] nor [99] mentions *F. orientalis*. The documented elevation ranges of these species extend from near sea level (*P. brutia* [81]) to about 2000–2200 m (*P. nigra* [83], *Q. cerris* [90], and *F. orientalis* [93]).

### 4.5. Climate Forcing

We obtained daily forcing from the ISIMIP3b bias-adjusted output [100] for its five primary CMIP6 GCMs (GFDL-ESM4, IPSL-CM6A-LR, MPI-ESM1-2-HR, MRI-ESM2-0 and UKESM1-0-LL) under both SSP1-2.6 and SSP5-8.5, and extracted the data at the six site locations. Before running, the forcing loader verifies that each GCM × scenario block is complete and discards incomplete blocks; otherwise, the missing values would carry over into the stomatal solution. We took CO_2_ concentrations from the SSP greenhouse gas concentration dataset of Meinshausen et al. [101]: in 2025, 427.7 μmol mol^−1^ under SSP1-2.6 and 431.8 μmol mol^−1^ under SSP5-8.5; in 2075, 471.0 and 801.7 μmol mol^−1^, respectively. C_a_ was kept at the 2025 level during the baseline period and at the 2075 level during the projection period. Appendix C gives the resulting changes in the climate of the growing season at the six sites (Table A14). Forcing data were generated for both scenarios. With the seasonal reset, SSP1-2.6 was simulated using only GFDL-ESM4, and with year-round soil water using all five GCMs (Section 2.10); all other ensemble results from the five GCMs refer to SSP5-8.5.

### 4.6. Experimental Design

Each 11-year window runs from 2020 to 2030 for the baseline and from 2070 to 2080 for the projection. The growing season is a fixed range of days of the year for each leaf habit: 60–330 for evergreen conifers, 60–300 for evergreen sclerophyll, and 105–286 for deciduous species. In the projection window, year *i* is matched with year *i* of the baseline window, so that a frozen channel is always compared to a particular baseline year. A cell represents one species at a single site under one GCM. In each of the four attribution runs (arms), the value used is one of the following from the paired baseline year: C_a_ (for the CO_2_ channel); the daily maximum and minimum temperatures together with the vapor pressure deficit (for the warming channel); precipitation (for the precipitation channel); or photosynthetically active radiation (for the radiation channel). The warming channel thus includes the effects of air temperature and the associated vapor pressure deficit, which are not separated. Since these four channels are the only forcing variables that differ between a scenario year and its baseline, the identity

projected change = CO_2_ + warming + precipitation + radiation + residual (1)

holds by construction, and the residual part is given separately rather than being allocated among the different channels. For CO_2_, a scalar quantity, freezing creates a difference within the same year; for the three weather channels, freezing replaces the paired baseline-year series and thus accounts for variability in both years. The four contributions were therefore averaged over the 11-year windows. The design includes 20,151 model–season simulations: 19,746 in 11-year, ten-year, or five-year windows and 405 single-year simulations, used solely to compare sampling units (Section 2.1 and Section 3.2). These simulations include a common historical baseline window (2005–2014, with atmospheric CO_2_ at 389 μmol mol^−1^) shared by both scenarios and four structural arms that alter growing-season length and soil texture (GFDL-ESM4, five-year windows); Section 2.8 presents both groups. They also include a set that ran *Q. coccifera* using the two alternative published stem P_50_ values (1980 simulations; Section 2.4). The tests in Section 2.9 and Section 2.10 add 30,240 model–season simulations from eight further sets of runs (Section 4.7).

### 4.7. Sensitivity Analyses

The reduction in *V*_cmax25_ and *J*_max25_ (the values at 25 °C) to reflect acclimation of the carboxylation capacity to growth CO_2_ was set at 6.6% and 2.5%, respectively, for every 100 μmol mol^−1^ above the scenario baseline (derived from the all-species means in [13], about twice the rate for trees [17]) and at twice those values. At 801.7 μmol mol^−1^, this corresponds to a 24% lower *V*_cmax25_ and a 9% lower *J*_max25_ (49% and 18% when doubled), which is beyond the CO_2_ range of the FACE experiments (475–600 μmol mol^−1^ [13]); the linear scaling beyond that range is an assumption. Because the scaling is referenced to each scenario’s baseline, the baseline window remains unaltered; we confirm this by showing an exact zero difference in all 1782 baseline runs, meaning only the elevated-CO_2_ end is affected. For the energy balance sensitivity, shortwave absorptance was set to 0.40 and 0.60, and boundary-layer conductance was multiplied by 0.7 and 1.4, using five-year windows (2023–2027 and 2073–2077). All variants use the same weather sequence, so comparisons control for differences in weather forcing; interannual variability remains within each sequence.

The tests in Section 2.9 used four further sets of runs under SSP5-8.5. (i) Year-round soil water: Each window was simulated as a chain of consecutive years on one soil column, starting at a relative soil moisture of 0.60 two years before the window; these two spin-up years are not reported. Between two growing seasons, the soil received precipitation and lost water through leakage and soil evaporation, with no transpiration. We ran both 11-year windows this way with the five GCMs. We also ran their five central years (2023–2027 and 2073–2077) as separate chains with their own spin-up years. In these chains, *A*_season_ differed from that of the same years of the 11-year chains by 0.04 gC m^−2^ on average and by more than 1 gC m^−2^ in 11 of 1350 seasons (at most 17.1 gC m^−2^). The GFDL-ESM4 chains of the central years are the reference for set (ii).

(ii) One assumption at a time: For the five central years of each window, with GFDL-ESM4 and year-round soil water, we made the following changes one at a time. We calculated stand water use as leaf transpiration multiplied by the sunlit leaf area index, [1 − exp(−0.5 LAI)]/0.5, where 0.5 is the extinction coefficient [73], rather than by the full LAI. Precipitation was multiplied by 1.3 and 1.6, and soil evaporation by 0.5 and 2. We made the air 3 and 6 °C cooler at the same relative humidity. The soil was loam or loamy sand. The leaf energy balance was solved without linearization, the soil water balance was stepped hourly instead of daily, the spin-up started at a relative soil moisture of 0.45 or 0.75, and the Weibull scale was corrected so that 50% of the conductance is lost at P_50_.

(iii) Attribution with the weather in sequence: For 2073–2077, with the five GCMs and the seasonal reset, we kept the scenario weather and removed the monthly mean change between 2020–2030 and 2070–2080 from one weather channel, or from all three with C_a_ at its baseline value. We shifted daily maximum and minimum temperatures and relative humidity by the monthly mean difference, then computed vapor pressure deficit from them. We scaled precipitation and shortwave radiation (from which we derived photosynthetically active radiation) by the ratio of monthly means.

(iv) Hydraulics switched off: We ran both 11-year windows with the five GCMs without the soil column and without water stress. In all four sets, the model also counted the days above thresholds 1–3 °C lower and higher than *T*_crit_. In addition, it counted days when a leaf with a stomatal conductance of 0.01 (the residual conductance of the stomatal model), 0.05, or 0.10 mol m^−2^ s^−1^ would exceed *T*_crit_ under the same forcing. These counts use only the energy balance and do not change the soil water. The four sets comprise 13,770 model–season simulations (16,362 with the spin-up seasons, which are not reported).

The analyses in Section 2.10 used four further sets of runs, all with year-round soil water and two spin-up years, and the counts of set (i). (v) Full factorial: For 2073–2077, with the five GCMs, we ran all 16 combinations of the four channels with and without their mean change, with C_a_ at its 2075 or 2025 value and the mean change of the weather channels removed as in set (iii). With *v*(*S*) denoting *A*_season_ when the channels in the set *S* have changed, the first-in contribution of channel *i* is *v*({*i*}) − *v*(∅), the last-in contribution is *v*(all) minus *v* of all channels except *i*, and the Shapley value is φ_i_ = Σ |*S*|!(3 − |*S*|)!/4! [*v*(*S* ∪ {*i*}) − *v*(*S*)], summed over the eight subsets *S* of the other three channels; the four Shapley values add up to *v*(all) − *v*(∅). (vi) The CO_2_ term: In the run with C_a_ at its baseline value, the leaf was recomputed every day with the scenario C_a_, first at the water stress of that run at every time step and then from its soil moisture at the start of the day. The first difference is the direct effect at unchanged water status, the second the adjustment of water status within the day, and the remainder of the CO_2_ term the effect of soil water carried forward from earlier days. Both direct calculations were repeated with the photorespiratory CO_2_ compensation point (Γ*) set to zero. (vii) Traits: With GFDL-ESM4 and the five central years of both windows, we changed one trait at a time for all species: rooting depth ×0.5 and ×1.5, LAI ×0.7 and ×1.3, *V*_cmax25_ ×0.7 and ×1.3 (with *J*_max25_ and dark respiration scaled with it), g_1_ of 3 and 5 kPa^0.5^, and the turgor loss point by ±0.5 MPa. (viii) SSP1-2.6: Both 11-year windows of SSP1-2.6 with the five GCMs. These four sets comprise 16,470 model–season simulations (22,410 with the spin-up seasons). The threshold variants were computed from the counts of set (i): a common threshold of *T* °C corresponds to the count at an offset of *T* − 46 °C from the assigned threshold for the conifers, *T* − 45 °C for the sclerophyll, and *T* − 44 °C for the deciduous species.

The analyses in Section 2.11 and Section 2.12 used the seasonal maxima of set (i), the leaf model at fixed conditions, and three further sets of runs, with year-round soil water and two spin-up years unless stated otherwise. (ix) Hourly leaf temperature: The 11-year chains of set (i) were repeated with additional output computed from the leaf and air temperatures of every hourly step; they reproduced the stored output of set (i) exactly. The new output comprises, for each day, the maximum leaf temperature and the number of hours at or above *T*_crit_ and, for each season, the hours at or above thresholds from *T*_crit_ − 3 to *T*_crit_ + 6 °C, the degree-hours above *T*_crit_, and the lit hours in 1 °C bins of leaf and air temperature. (x) Chloroplast-basis constants: With the Rubisco constants estimated on a chloroplast basis (Appendix D) and the five GCMs, we repeated the five-year chains of set (i) and the two runs of set (v) without the CO_2_ change and without the warming, and, with the seasonal reset, the runs of Section 2.2 for 2023–2027 and 2073–2077 and the two runs for 2073–2077 with C_a_ and with the warming channel at their baseline (Section 4.6). (xi) Implementation variants. With GFDL-ESM4, we repeated the five-year chains of set (i) with the PAR curve over the astronomical day, with the transpiration charged to the soil computed with the boundary-layer conductance in series, and with all five changes together: these two, the chloroplast-basis constants, the Weibull scale of set (ii), and, for *Pinus nigra*, the shape *c* = 4.40 (Appendix D). All five changes were also run with the other four GCMs, and the shape of *P. nigra* alone with the five GCMs. These three sets comprise 10,510 model–season simulations (12,986 with the spin-up seasons). For the variability of the thresholds among leaves, the threshold of a leaf was taken as *T*_crit_ + *x*, with *x* normally distributed with mean 0 and standard deviation σ (2.38 or 2.87 °C, the standard deviations of the individual measurements of the two species [12]), and the expected number of days per season was *E* = Σ_d_ Φ[(*T*_d_ − *T*_crit_)/σ], where *T*_d_ is the maximum leaf temperature of day *d* of the season and Φ the standard normal distribution function. For acclimation, the threshold of each species, site, and GCM in 2070–2080 was raised by 0.2 or 0.4 times the change in the mean temperature of its growing season between the two windows. *T*_50_ was taken as *T*_crit_ + 5.32 or + 6.50 °C, the mean paired differences in the source data [12]. The leaf at fixed conditions (Table A11) was solved with the model’s leaf routine for *Pinus brutia*, *Quercus cerris*, and *Q. coccifera* at an intercellular CO_2_ of 0.766 C_a_, the ratio given by Equation (A9) at a vapor pressure deficit of 1.5 kPa when the residual conductance is neglected, without water stress, at leaf temperatures of 20–35 °C and a PAR of 1800 and 300 μmol m^−2^ s^−1^; the leaf temperature at which net assimilation is zero was solved at C_i_ = C_a_ and a PAR of 2000 μmol m^−2^ s^−1^.

### 4.8. Software, Data and Reproducibility

The model was implemented in Python 3.11 using NumPy and SciPy. The reported implementation passed 304 tests across 13 modules; the energy balance and trait table were tested only indirectly. Passing these tests establishes consistency with the tested implementation requirements, not independent validation of physiological predictions. The source code, analysis scripts, and result tables are available upon request from the corresponding author (Data Availability Statement). ISIMIP3b forcing must be obtained separately from the ISIMIP repository under its license terms [100]. The empirical comparisons in Section 2.7 are plausibility checks rather than calibration or independent validation.

### 4.9. Statistics

In Section 2.2, Section 2.3 and Section 2.5, the values given as mean ± SE are descriptive standard errors of the arithmetic means across cells, calculated under an independence assumption; they do not measure cell dispersion or account for shared sites and GCMs. Since the cells are not independent, the confidence interval for the projection (Section 2.6, Table 5) was calculated using a model that includes a fixed intercept and has species, site and GCM as crossed random effects, according to the equation y_ijk_ = μ + s_i_ + t_j_ + g_k_ + e_ijk_, where s_i_, t_j_ and g_k_ represent the species, site and GCM effects respectively and e_ijk_ is the unexplained variation, each of which is normally distributed with its own variance. The variance components were estimated by means of REML, by minimizing −2lᵣ = log|V| + log|X′V^−1^X| + r′V^−1^r over the logarithms of the four variances using the Nelder–Mead algorithm, with the matrix V being constructed densely and then factorized by means of Cholesky factorization. The standard error of the mean is (X′V^−1^X)^−1^ᐟ^2^, and the 95% confidence interval is the GLS mean ± 1.96 standard errors; since there are few levels for each random factor, this Wald interval is only an approximation and may be too narrow. For comparison, we also provide an interval based on the five GCM means with four degrees of freedom; where the intervals differ, we report the wider one. The implementation (varcomp.py in the code package) requires only NumPy and SciPy. We obtained the species-level values in Table 3 and Table A1 and Figure 5 by fitting the same model to the site × GCM cells for each species, with site and GCM treated as random effects. As the cells for each species form a complete grid, the GLS mean is equal to the arithmetic mean, and it is only the standard error that changes; in the case of *Picea orientalis*, which is recorded at only one site, the site term cannot be estimated, and its standard errors are therefore lower bounds. The same model was fitted to the Shapley values, the SSP1-2.6 net change, and the scenario difference in Section 2.10. All of these intervals are conditional on the sampled species, sites, and GCMs: the five GCMs were bias-adjusted to the same observation-based dataset and follow the same CO_2_ pathway, so they are not independent samples of climate models, and the intervals are not prediction intervals for other forests, climate models, or model structures.

### 4.10. Use of Generative Artificial Intelligence

The authors designed the study, implemented and executed the model and analyses, and made the scientific decisions and interpretations. Claude (Anthropic) was used to assist with English editing and language polishing, to review the model implementation and analysis and figure scripts, and to help locate distribution literature for Table 8. The authors checked the numerical results against the simulation outputs and independently verified the cited sources. AI-assisted review was not treated as independent model validation. The authors reviewed and revised the assisted material and take responsibility for the scientific content, code, results, and final wording.

## 5. Conclusions

For eight Anatolian tree species across 27 species–site combinations at six locations, the coupled leaf model projected a positive ensemble mean change in seasonal net assimilation under the SSP5-8.5 scenario between the years 2020–2030 and 2070–2080. With year-round soil water, the mean increase was 106.5 gC m^−2^ of leaf area, with an approximate 95% crossed random-effects interval of +43 to +167. All five GCM means were positive, although 11 of the 135 cells showed a decline. Under SSP1-2.6, the five-GCM increase was +33.6 gC m^−2^, and 25 cells declined. When soil moisture was reset at the start of each season, the increase was 130.3 gC/m^2^, with an approximate interval of +76 to +187 gC m^−2^. In that configuration, the CO_2_ contribution (+219.5 gC m^−2^) exceeded the negative contributions of temperature–VPD (-66.7), precipitation (-38.8), and radiation (-2.8), with a residual of +19.1. With year-round soil water, a full factorial attribution kept the signs and order of the four contributions (Shapley values: CO_2_, +218.3; warming, −63.2; precipitation, −41.4; radiation, −1.9 gC m^−2^), and about one-third of the CO_2_ contribution came from water saved by lower stomatal conductance. The reported photorespiratory fraction declined from 14.6% to 9.3%. Species and sites accounted for more fitted variance than GCMs within this ensemble. However, neither the variance partition nor its confidence intervals included the full uncertainty associated with the assumed traits, canopy structure, nutrient limitation, or sink feedbacks. In forest FACE experiments, less of the leaf-level gain reached stand growth, so the modeled gain is an upper bound for stand productivity.

Modeling carbon gain and thermal exposure provided different assessments of leaf responses. Days above the assigned photosystem II thresholds increased from approximately 1.5 to 12.2 per season across the five GCMs, primarily among broadleaved species. With thresholds that vary among leaves as in the source measurements, or that acclimate to the warming, the number in the 2070s was 6.5–16.5 days, at least four times that of the 2020s; on a day above its threshold, the leaf stayed above it for 3.4 h on average in the 2070s. With a common threshold of 44 °C for all species, they increased from 1.8 to 16.9, and the broadleaved species still exceeded it about four times as often as the conifers. These counts indicate modeled threshold exposure, not actual photosystem damage, mortality, or forest resilience. Their magnitude depended strongly on assigned thresholds, energy balance parameters, stomatal behavior, and air temperature at the modeled locations. The positive mean assimilation change persisted under the tested photosynthetic acclimation rates, growing-season adjustments, and soil textures, but was more sensitive to soil-water representation and canopy water withdrawal. These tests support the direction of the ensemble mean response within the examined configurations. However, they do not establish the response’s robustness to all omitted processes. Since the model represents a sunlit leaf, its outputs cannot be interpreted as stand productivity. Priorities for further development include canopy integration, species-specific trait measurements, elevation-adjusted forcing, and independent leaf-flux and temperature measurements.

## Figures and Tables

**Table 1:** The attribution of forcing-channel effects under SSP5-8.5 for the period 2070–2080 in comparison with 2020–2030 (in gC m^−2^ of leaf area per season). *n* refers to the number of cells per GCM; the ensemble row includes the five GCMs (5 × 27 cells). The ± figures are standard errors across the cells and do not indicate projection uncertainty.

GCM	*n*	CO_2_	Warming	Precip.	Radiation	Residual	Net Change
GFDL-ESM4	27	+220.2	−52.0	−39.3	−0.9	+14.4	+142.4 ± 9.8
IPSL-CM6A-LR	27	+222.9	−83.8	−20.8	−3.3	+12.8	+127.7 ± 13.4
MPI-ESM1-2-HR	27	+211.3	−60.3	−69.1	−3.0	+28.0	+107.0 ± 13.0
MRI-ESM2-0	27	+215.1	−68.7	−41.7	−4.7	+27.9	+127.9 ± 11.7
UKESM1-0-LL	27	+227.8	−68.4	−23.2	−2.0	+12.3	+146.5 ± 14.4
Ensemble	135	+219.5 ± 5.2	−66.7 ± 3.1	−38.8 ± 3.6	−2.8 ± 0.4	+19.1 ± 2.6	+130.3 ± 5.7

**Table 2:** The scenario contrast is given for GFDL-ESM4 (27 cells), while the final row presents the five-GCM ensemble under SSP5-8.5 (135 cells). The channel contributions and the net change are expressed in gC m^−2^ per season (mean ± SE). The number of days above *T*_crit_ is the number of days per season when peak leaf temperature surpassed the critical photosystem II temperature. The SSP1-2.6 entry of the last row comes from the runs with year-round soil water (Section 2.10); the number of days does not depend on the soil water configuration (Section 2.9).

Quantity	SSP1-2.6	SSP5-8.5
CO_2_	+33.6 ± 1.7	+220.2 ± 11.5
Warming	+5.0 ± 2.5	−52.0 ± 6.1
Precipitation	+17.2 ± 5.0	−39.3 ± 4.6
Radiation	−2.3 ± 0.8	−0.9 ± 0.4
Residual	+3.4 ± 2.4	+14.4 ± 5.0
Net change	+56.9 ± 6.5	+142.4 ± 9.8
Baseline *A*_season_ (gC m^−2^), 2020s	433.3	450.5
Days above *T*_crit_, 2020s → 2070s	1.6 → 2.0 (1.3×)	1.0 → 7.2 (7.3×)
Days above *T*_crit_, five-GCM ensemble	1.8 → 2.8 (year-round soil water; Table A9)	1.5 → 12.2; range 5.2–17.5

**Table 3:** The forcing-channel attribution by species under SSP5-8.5 (gC m^−2^ per season). The values are the means, averaged over the species’ sites × GCM cells ± standard errors, from a model in which site and GCM are treated as crossed random effects (Section 4.9); *n* refers to the number of cells. The net change is the sum of the four channels and the residual (Equation (1)); the residual is not shown.

Species	*n*	CO_2_	Warming	Precipitation	Radiation	Net Change
*Quercus coccifera*	15	+286.4 ± 11.6	−120.7 ± 10.4	−43.7 ± 15.3	−6.7 ± 1.7	+153.2 ± 9.9
*Juniperus excelsa*	25	+285.8 ± 6.4	−27.6 ± 15.4	−25.0 ± 9.0	+0.5 ± 0.7	+214.1 ± 6.5
*Picea orientalis*	5	+261.1 ± 6.1	−19.3 ± 13.3	−30.9 ± 20.5	−4.9 ± 3.2	+192.3 ± 18.2
*Cedrus libani*	15	+246.0 ± 4.3	−74.7 ± 4.6	−46.7 ± 20.0	−0.9 ± 0.4	+158.6 ± 17.0
*Pinus brutia*	15	+198.8 ± 12.3	−64.2 ± 8.5	−57.1 ± 21.4	−1.1 ± 0.7	+111.0 ± 10.9
*Pinus nigra* subsp. *pallasiana*	25	+197.6 ± 7.8	−76.5 ± 8.0	−56.1 ± 14.5	−1.9 ± 0.6	+98.5 ± 11.4
*Fagus orientalis*	10	+164.8 ± 85.0	−58.9 ± 12.7	−41.9 ± 12.9	−8.5 ± 4.1	+74.9 ± 71.9
*Quercus cerris*	25	+144.9 ± 5.0	−72.7 ± 7.5	−17.1 ± 8.2	−4.0 ± 1.3	+68.9 ± 9.4

The standard errors account for cells that share a site or a GCM; since there are five GCMs and at most five sites per species, the errors are only approximate. Eight of the values are within two standard errors of zero: the warming and radiation terms for *Juniperus excelsa*, the latter being near zero; the radiation term for *Pinus brutia*; the warming, precipitation, and radiation terms for *Picea orientalis*, which is found at only one site, so that site-to-site variation cannot be estimated and hence its standard errors represent lower bounds; and the CO_2_ term and the net change for *Fagus orientalis* because its two sites differ by more than a factor of three in the CO_2_ term (+80 gC m^−2^ at Balıkesir and +250 gC m^−2^ at Rize).

**Table 4:** Sensitivity to the energy balance parameters (GFDL-ESM4, SSP5-8.5, 27 cells, five-year windows: 2023–2027 and 2073–2077). The peak ΔT refers to the average of the seasonal maximum values of the leaf–air temperature difference from 2073 to 2077. The net change is the change in seasonal net assimilation between the two windows.

Parameter Set	Peak Δ*T* (°C)	Days Above *T*_crit_, 2020s	Days Above *T*_crit_, 2070s	*A*_season_ 2070s (gC m^−2^)	Net Change (gC m^−2^)
*a*_sw_ 0.40	3.74	0.09	2.76	642.3	+162.2
*g*_b_ × 1.4	3.79	0.10	3.07	625.1	+159.3
default (*a*_sw_ 0.50, *g*_b_ × 1.0)	5.13	0.73	7.36	606.6	+156.2
*a*_sw_ 0.60	6.54	3.40	14.36	572.8	+150.0
*g*_b_ × 0.7	6.98	4.17	15.61	581.5	+151.8

**Table 5:** The variance components of the projected change in Aseason under SSP5-8.5, as estimated by REML with species, site, and GCM treated as crossed random effects (for 135 cells), are given; the percentages relate to the total variance, “Resid. %” denotes the variance which is not accounted for by species, site, or GCM, and the “Residual term” entry is the residual from Equation (1). The 95% interval is calculated as the GLS mean ± 1.96 standard errors. The GLS means differ slightly from the arithmetic means in Table 1 because not every species occurs at every site; each row is fitted separately, so the GLS means of the channels and the residual need not add up to that of the net change. These are approximate confidence intervals for conditional ensemble means, not prediction intervals for individual species–site combinations.

Quantity	GLS Mean	Site %	Species %	GCM %	Resid. %	95% Interval (gC m^−2^)
Net change	+131.5	31	50	3.1	16	+76 to +187
CO_2_ channel	+218.9	43	56	0.3	1	+134 to +304
Warming channel	−64.1	4	60	7.6	28	−89 to −39
Precipitation channel	−40.0	7	11	17.9	63	−62 to −18
Radiation channel	−3.5	32	33	7.6	28	−6.7 to −0.3
Residual term	+18.4	1	49	5.0	45	+1 to +36

**Table 7:** Tests of architectural assumptions. For the baseline block, the entire ensemble (135 cells; 11-year windows, except for the ten-year common baseline) is used; for the growing-season and soil blocks, GFDL-ESM4 (27 cells, five-year windows) is used. Each block is compared with its own default, and the absolute values are not comparable across the blocks.

Assumption	Variant	Net Change (gC m^−2^)	Change vs. Default
Baseline window	own, 2020–2030 (default)	+130.3	—
	common, 2005–2014	+153.3	+23.0 (+17.7%)
Growing season	fixed calendar (default)	+156.2	—
	+15 days	+160.9	+4.6 (+3.0%)
	−15 days	+151.2	−5.1 (−3.3%)
Soil texture	sandy loam (default)	+156.2	—
	loam	+145.3	−10.9 (−7.0%)
	loamy sand	+161.4	+5.1 (+3.3%)

## Data Availability

The source code for the model, the full simulation output (including 60,901 model–season simulations), the analysis script that can regenerate each number, table, and figure in this paper, and the derived result tables are available upon request from the corresponding author. The bias-adjusted climate forcing data from ISIMIP3b are available from the ISIMIP repository, subject to its license terms.

## Abbreviations

The following abbreviations are used in this manuscript:

*A*_season_	Seasonal net assimilation per unit leaf area
A/E	Water-use efficiency, carbon assimilated per unit water transpired
*a*_sw_	Shortwave absorptance
C_a_	Atmospheric CO_2_ mole fraction
C_i_	Intercellular CO_2_ mole fraction
CMIP6	Coupled Model Intercomparison Project Phase 6
FACE	Free-air CO_2_ enrichment
FvCB	Farquhar–von Caemmerer–Berry photosynthesis model
*g*_b_	Boundary-layer conductance
GCM	General circulation model
GLS	Generalized least squares
GPP	Gross primary productivity
ISIMIP3b	Inter-Sectoral Impact Model Intercomparison Project, phase 3b
*J*_max25_	Maximum electron transport rate at 25 °C
LAI	Leaf area index
NEP	Net ecosystem productivity
NPP	Net primary productivity
P_50_	Water potential at 50% loss of hydraulic conductivity
PAR	Photosynthetically active radiation
RCP	Representative Concentration Pathway
REML	Restricted maximum likelihood
SE	Standard error
SSP	Shared Socioeconomic Pathway
*T*_50_	Temperature at which the photosystem II efficiency falls by 50%
*T*_crit_	Critical leaf temperature for photosystem II
*V*_cmax25_	Maximum carboxylation rate at 25 °C
VPD	Vapor pressure deficit

## Appendix A. Trait Provenance

Out of the 48 trait values in the trait table of the model (which consists of eight species and six parameters: P_50_, turgor loss point, minimum conductance, maximum plant conductance, rooting depth and Vcmax25), 2 (4%) were actually measured for the species in question, 13 are surrogates taken from a named congener, and 33 have been assumed (Table A16). The trait table records the minimum conductance, but the simulations reported here do not use it; instead, they use the stomatal model’s residual conductance as the lower limit (Section 4.1). The results therefore depend on 40 of the 48 values: two measured, eight surrogates, and 30 assumed. Each value includes a provenance flag and source in the code, from which the coverage table can be generated. The maximum plant conductances are counted as assumed for all species because the values used for four of them are not reported as such in the sources recorded in the code. The two measured values are the P_50_ of *Cedrus libani* and *Quercus cerris* (for *Q. cerris*, the water potential at 40% loss of petiole conductivity in seedlings); the P_50_ of *Juniperus excelsa* was measured on *J. seravschanica* and is counted as a surrogate, and the P_50_ of *Fagus orientalis* is counted as assumed because it differs from the values measured on its congener (Table A16); rooting depth is assumed for all eight species, and the carboxylation capacity is assumed for seven species and taken from *Q. ilex* for *Q. coccifera*. LAI is not among the six parameters; it has been measured only for *C. libani*, and the value used lies within the measured range (Table A16). We make this coverage explicit because it limits the confidence we can place in the species-level results. One conflict in the literature remains unresolved: the surrogate stem P_50_ values compiled for *Q. ilex* and applied to *Q. coccifera* range from −3.88 to −7.13 MPa, a range that may reflect measurement artifacts in long-vesselled species as well as differences among studies. The main runs use the most negative value, which lies within 0.2 MPa of the −6.96 MPa reported for *Q. coccifera* itself [15]. Because *Q. coccifera* has the largest CO_2_ contribution in Table 3, we reran it using the other two values (Section 2.4, Table A1); its net change differed by less than 0.3 gC m^−2^. No rank in Table 3 was affected, as stomatal closure at the turgor loss point keeps leaf water potential well above all three values. Furthermore, we revised the turgor loss points for the oaks (to −1.9 MPa for *Q. cerris* and −2.6 MPa for *Q. coccifera*) following Kullberg et al. [12], whose data give treatment means of −1.57 to −2.10 MPa for *Q. pubescens*, measured by Mas et al. [102]; the previously used values (−3.0 and −3.3 MPa) were considerably more negative than this measurement.

**Table A1:** The three published stem P_50_ values of *Q. ilex*, applied to *Quercus coccifera* (SSP5-8.5; three sites × five GCMs; gC m^−2^ per season). The channel values are means across the 15 site × GCM cells, with standard errors within 0.1 of those in Table 3 (11.6, 10.4, 15.3 and 1.7) in each row; the net change is the mean ± SE from the same model. The ranks are based on the eight species (for CO_2_ and net change, ranked from largest to smallest; for warming, ranked from highest cost to smallest). The final column shows the ensemble net change (for 135 cells) obtained by running the cells of this species at the specified P_50_.

Stem P_50_ (MPa)	CO_2_	Warming	Precip.	Radiation	Net Change	Rank (CO_2_, Warming, Net)	Ensemble Net
−7.13 (default)	+286.4	−120.7	−43.7	−6.7	+153.2 ± 9.9	1, 1, 4	+130.3
−4.66	+286.3	−120.8	−43.7	−6.7	+153.1 ± 9.9	1, 1, 4	+130.3
−3.88	+286.2	−120.8	−43.7	−6.7	+153.0 ± 9.9	1, 1, 4	+130.3

## Appendix B. Results of the Tests in Section 2.9 and Section 2.10

Table A2, Table A3, Table A4 and Table A5 give the results of the tests reported in Section 2.9, Table A6, Table A7, Table A8, Table A9 and Table A10 those of Section 2.10, Table A11 and Table A12 the leaf at fixed conditions and the hourly leaf temperatures of Section 2.11, and Table A13 the tests of Section 2.12; the runs and calculations are described in Section 4.7.

**Table A2:** The net change in *A*_season_ (gC m^−2^ per season) under SSP5-8.5 with the seasonal reset, as in Section 2.1, Section 2.2, Section 2.3, Section 2.4, Section 2.5, Section 2.6, Section 2.7 and Section 2.8, and with year-round soil water (five GCMs, 11-year windows). The values are means ± SE across the *n* cells of each row; for the species, these standard errors can therefore differ from the model-based ones in Table 3. Relative soil moisture is the value at the start of the season in runs with year-round soil water. Water use is stand-level (leaf transpiration multiplied by LAI), expressed as a percentage of annual precipitation. The last three columns give the values for 2020–2030 and 2070–2080.

Group		*n*	Net Change, Seasonal Reset	Net Change, Year-Round	Relative Soil Moisture at Season Start	Water Use, Seasonal Reset (%)	Water Use, Year-Round (%)
All cells		135	+130.3 ± 5.7	+106.5 ± 6.4	0.58 → 0.56	57 → 62	54 → 57
GCM	GFDL-ESM4	27	+142.4 ± 9.8	+117.3 ± 11.7	0.59 → 0.56	55 → 61	53 → 56
	IPSL-CM6A-LR	27	+127.7 ± 13.4	+89.4 ± 16.6	0.60 → 0.54	55 → 65	52 → 58
	MPI-ESM1-2-HR	27	+107.0 ± 13.0	+84.3 ± 14.4	0.56 → 0.55	59 → 62	55 → 56
	MRI-ESM2-0	27	+127.9 ± 11.7	+108.3 ± 12.4	0.59 → 0.56	57 → 62	54 → 57
	UKESM1-0-LL	27	+146.5 ± 14.4	+133.4 ± 14.6	0.58 → 0.57	58 → 59	54 → 55
Site	Antalya	30	+133.3 ± 10.7	+131.2 ± 10.6	0.63 → 0.62	41 → 43	41 → 43
	Balıkesir	30	+100.9 ± 12.7	+81.7 ± 12.5	0.59 → 0.57	56 → 61	55 → 59
	Konya	20	+144.8 ± 15.0	+87.3 ± 17.8	0.52 → 0.47	76 → 85	69 → 73
	Eskişehir	15	+111.5 ± 17.8	+51.0 ± 21.6	0.46 → 0.42	89 → 96	75 → 76
	Kahramanmaraş	30	+143.7 ± 11.1	+126.3 ± 11.7	0.61 → 0.58	52 → 59	51 → 56
	Rize	10	+168.8 ± 16.6	+169.4 ± 16.4	0.64 → 0.64	33 → 29	33 → 29
Species	*Quercus coccifera*	15	+153.2 ± 8.1	+102.1 ± 13.3	0.58 → 0.54	74 → 82	71 → 75
	*Juniperus excelsa*	25	+214.1 ± 4.6	+185.2 ± 10.4	0.56 → 0.53	56 → 58	53 → 54
	*Picea orientalis*	5	+192.3 ± 18.2	+192.2 ± 18.2	0.65 → 0.65	34 → 29	34 → 29
	*Cedrus libani*	15	+158.6 ± 11.7	+137.8 ± 14.7	0.59 → 0.56	53 → 57	51 → 54
	*Pinus brutia*	15	+111.0 ± 10.9	+110.4 ± 10.7	0.62 → 0.61	37 → 39	37 → 39
	*Pinus nigra* subsp. *pallasiana*	25	+98.5 ± 8.2	+82.7 ± 10.1	0.57 → 0.55	57 → 61	55 → 58
	*Fagus orientalis*	10	+74.9 ± 26.7	+74.3 ± 27.2	0.61 → 0.61	42 → 45	42 → 44
	*Quercus cerris*	25	+68.9 ± 5.3	+29.0 ± 9.1	0.56 → 0.52	72 → 83	64 → 70

**Table A3:** One assumption changed at a time, with year-round soil water (GFDL-ESM4, SSP5-8.5, 27 cells; five-year windows: 2023–2027 and 2073–2077). The net change is the change in *A*_season_ between the two windows (gC m^−2^ per season, mean across cells). The change vs. the reference (gC m^−2^ per season; in parentheses, as a percentage of the reference) is the mean of the differences per cell so that it may differ by 0.1 from the difference between the rounded net changes. The second row is the configuration of Section 2.1, Section 2.2, Section 2.3, Section 2.4, Section 2.5, Section 2.6, Section 2.7 and Section 2.8 for the same cells and years. The last two columns give the values for 2023–2027 and 2073–2077. The last four rows remove the simplifications of the implementation (Section 2.12; Appendix D): The PAR curve spans the astronomical day, the transpiration charged to the soil includes the boundary-layer conductance, and the Rubisco constants are those estimated on a chloroplast basis; the last row combines these three changes with the corrected scale of the vulnerability curve and, for *Pinus nigra*, the shape of its measured curve (*c* = 4.40).

Assumption	Net Change (gC m^−2^)	Change vs. Reference	Days Above *T*_crit_	Relative Soil Moisture at Season Start
Year-round soil water (reference)	+132.4	—	0.73 → 7.38	0.59 → 0.56
Seasonal reset (Section 2.1, Section 2.2, Section 2.3, Section 2.4, Section 2.5, Section 2.6, Section 2.7 and Section 2.8)	+156.2	+23.9 (+18.0%)	0.73 → 7.36	0.60 (set)
Only the sunlit leaf area withdraws soil water	+198.8	+66.5 (+50.2%)	0.73 → 7.00	0.60 → 0.58
Precipitation × 1.3	+152.1	+19.7 (+14.9%)	0.73 → 7.36	0.62 → 0.60
Precipitation × 1.6	+164.3	+31.9 (+24.1%)	0.73 → 7.34	0.64 → 0.62
Soil evaporation × 0.5	+144.9	+12.6 (+9.5%)	0.73 → 7.36	0.60 → 0.58
Soil evaporation × 2	+108.2	−24.1 (−18.2%)	0.73 → 7.38	0.56 → 0.54
Air 3 °C cooler	+137.5	+5.1 (+3.8%)	0.02 → 1.59	0.59 → 0.57
Air 6 °C cooler	+140.4	+8.1 (+6.1%)	0.00 → 0.24	0.59 → 0.57
Soil texture: loam	+127.3	−5.1 (−3.9%)	0.73 → 7.37	0.67 → 0.64
Soil texture: loamy sand	+134.3	+1.9 (+1.4%)	0.73 → 7.37	0.55 → 0.52
Energy balance without the linearization	+132.6	+0.2 (+0.1%)	0.66 → 7.17	0.59 → 0.56
Soil water balance stepped hourly	+132.3	−0.1 (−0.1%)	0.73 → 7.38	0.59 → 0.56
Spin-up started at 0.45	+132.4	0.0 (0.0%)	0.73 → 7.38	0.59 → 0.56
Spin-up started at 0.75	+132.4	0.0 (0.0%)	0.73 → 7.38	0.59 → 0.56
Vulnerability curve with 50% loss at P_50_	+132.4	0.0 (0.0%)	0.73 → 7.38	0.59 → 0.56
PAR curve over the astronomical day	+138.7	+6.3 (+4.8%)	0.79 → 7.46	0.59 → 0.56
Transpiration with the boundary-layer conductance	+129.6	−2.8 (−2.1%)	0.73 → 7.36	0.59 → 0.56
Rubisco constants on a chloroplast basis	+136.1	+3.7 (+2.8%)	0.73 → 7.36	0.59 → 0.56
All five changes together	+140.1	+7.7 (+5.8%)	0.79 → 7.46	0.59 → 0.56

**Table A4:** The attribution for 2073–2077 relative to 2023–2027 with two treatments of the weather channels (SSP5-8.5, five GCMs, 135 cells, seasonal reset; gC m^−2^ per season, mean ± SE across cells). Channel held at the baseline year: The channel is taken from the paired baseline year, as in Section 2.2 and Section 4.6. Weather in sequence: We keep the weather of 2073–2077 and remove the monthly mean change in the channel between 2020–2030 and 2070–2080. The CO_2_ term comes from the same runs in both cases. The residual is the net change minus the four terms (Equation (1)). With the weather in sequence, it has two parts: the non-additive part of the four mean changes, which is the change caused by the four together minus the sum of the four terms, and the weather sequence term, which is *A*_season_ of 2073–2077 with all four mean changes removed minus that of 2023–2027. Because of rounding, the sum of the terms in a column may differ from the net change by 0.1. The last three rows give the number of days above *T*_crit_ per season in 2073–2077 in the scenario run and in the runs without the change in temperature or in precipitation; the number was 1.48 in 2023–2027.

Term	Channel Held at the Baseline Year	Weather in Sequence	Range Among GCMs, Weather in Sequence
CO_2_	+219.4 ± 5.2	+219.4 ± 5.2	+215.6 to +223.9
Warming	−63.8 ± 3.2	−59.4 ± 3.3	−81.0 to −43.4
Precipitation	−40.1 ± 3.9	−29.7 ± 2.9	−57.5 to −14.2
Radiation	−3.1 ± 0.4	−1.8 ± 0.3	−2.7 to 0.0
Residual	+19.0 ± 2.7	+2.8 ± 3.7	−21.7 to +24.2
Residual: non-additive part	—	+6.3 ± 2.5	+1.4 to +9.6
Residual: weather sequence term	—	−3.5 ± 2.6	−25.0 to +14.6
Net change	+131.3 ± 6.3	+131.3 ± 6.3	+114.0 to +156.2
Days above *T*_crit_, scenario run	12.02	12.02	4.61 to 18.87
Days above *T*_crit_ without the change in temperature	1.35	1.59	0.96 to 1.93
Days above *T*_crit_ without the change in precipitation	11.99	12.01	4.60 to 18.85

**Table A5:** The number of days above *T*_crit_ per season under SSP5-8.5 (five GCMs, 11-year windows, means across cells). The first row and the rows with a shifted threshold or conductance held at a given value come from runs with year-round soil water; these counts are calculated using the leaf energy balance under the forcing of these runs. The last row counts the days on which the air temperature itself exceeded *T*_crit_. With the seasonal reset, the numbers modeled were 1.45 and 12.17. In the runs with hydraulics switched off, *A*_season_ was 832.4 gC m^−2^ in 2020–2030 and 1048.0 gC m^−2^ in 2070–2080.

Variant	All Cells, 2020–2030	All Cells, 2070–2080	Conifers, 2070–2080	Broadleaved, 2070–2080
As modeled (year-round soil water)	1.45	12.18	2.38	28.84
*T*_crit_ − 3 °C	7.73	31.39	13.34	62.08
*T*_crit_ − 2 °C	4.79	23.83	8.15	50.50
*T*_crit_ − 1 °C	2.78	17.46	4.72	39.13
*T*_crit_ + 1 °C	0.71	8.08	1.10	19.96
*T*_crit_ + 2 °C	0.28	5.11	0.47	12.99
*T*_crit_ + 3 °C	0.10	3.09	0.27	7.87
Hydraulics switched off	1.45	11.48	2.38	26.96
Conductance kept at the residual value (0.01 mol m^−2^ s^−1^)	1.45	12.24	2.38	29.00
Conductance kept at 0.05 mol m^−2^ s^−1^	0.29	6.05	1.13	14.41
Conductance kept at 0.10 mol m^−2^ s^−1^	0.02	2.22	0.44	5.25
Air temperature above *T*_crit_	0.00	0.46	0.26	0.79

**Table A6:** The attribution with year-round soil water and the full factorial design (2073–2077 relative to 2023–2027; SSP5-8.5, five GCMs, 135 cells; gC m^−2^ per season, mean ± SE across cells). First-in is the change caused by the channel alone; last-in is the full change minus the change without the channel, as in Section 2.2 but with the weather in sequence; the Shapley value is the average over all orders of changing the four channels (Section 4.7). The 95% interval is that of the crossed random-effects model (Section 4.9) for the Shapley value. The non-additive part is the change caused by the four mean changes together minus the sum of the four contributions of the column; for the Shapley values, it is zero by construction. The net change adds the weather sequence term (Section 2.9). The largest two-way interaction was −5.5 gC m^−2^ (CO_2_ × precipitation).

Term	First-In	Last-In	Shapley Value	Shapley Value, Range Among GCMs	95% Interval, Shapley Value
CO_2_	+222.5 ± 4.9	+212.8 ± 5.6	+218.3 ± 5.2	+212.7 to +222.9	+132 to +301
Warming	−60.5 ± 2.0	−67.1 ± 3.1	−63.2 ± 2.5	−86.3 to −47.8	−85 to −38
Precipitation	−36.2 ± 2.6	−47.8 ± 3.6	−41.4 ± 3.0	−61.7 to −18.8	−69 to −17
Radiation	−1.8 ± 0.2	−2.1 ± 0.3	−1.9 ± 0.2	−2.9 to −0.2	−4.0 to −0.2
Sum of the four	+124.0	+95.9	+111.9	—	—
Non-additive part	−12.1 ± 2.5	+16.0 ± 2.5	0	—	—
Change caused by the four together	+111.9 ± 6.5	+111.9 ± 6.5	+111.9 ± 6.5	—	—
Weather sequence term	−2.8 ± 2.8	−2.8 ± 2.8	−2.8 ± 2.8	—	—
Net change	+109.1 ± 7.1	+109.1 ± 7.1	+109.1 ± 7.1	—	—

**Table A7:** The CO_2_ term with year-round soil water split into three parts (2073–2077 relative to the run without the CO_2_ change; SSP5-8.5, five GCMs; gC m^−2^ per season, means across the *n* cells; all cells, mean ± SE). The CO_2_ term is the last-in term of Table A6. Direct effect: The scenario C_a_ at the water stress of the run without the CO_2_ change. Within the day: The adjustment of water status within the day. Carried forward: Soil water saved on earlier days (Section 4.7). The last column gives the direct effect with the photorespiratory CO_2_ compensation point (Γ*) set to zero.

Group	*n*	CO_2_ Term	Direct Effect	Within the Day	Carried Forward	Direct Effect, Γ* = 0
Antalya	30	+227.4	+174.7	−9.1	+61.8	+146.5
Balıkesir	30	+207.3	+145.4	−5.0	+66.8	+117.0
Konya	20	+203.3	+122.4	+1.9	+79.1	+97.8
Eskişehir	15	+189.0	+117.1	+0.8	+71.1	+96.3
Kahramanmaraş	30	+207.5	+135.9	−1.8	+73.4	+107.3
Rize	10	+256.4	+214.2	−4.8	+47.1	+172.6
Conifers	85	+234.5	+164.8	−1.4	+71.1	+131.2
Broadleaved species	50	+176.1	+120.4	−7.1	+62.7	+101.9
All cells	135	+212.8 ± 5.6	+148.4 ± 5.1	−3.5 ± 0.5	+68.0 ± 2.5	+120.4 ± 4.4

**Table A8:** One trait changed at a time for all species, with year-round soil water (GFDL-ESM4, SSP5-8.5, 27 cells; five-year windows: 2023–2027 and 2073–2077). The net change is the change in *A*_season_ between the two windows (gC m^−2^ per season, mean ± SE across cells); the change vs. the reference is given as a percentage of the reference. Changes in *V*_cmax25_ scale *J*_max25_ and dark respiration with it.

Variant	Net Change (gC m^−2^)	Change vs. Reference (%)	Cells with a Decline	Days Above *T*_crit_, 2023–2027 → 2073–2077
Reference (year-round soil water)	+132.4 ± 12.5	—	0	0.73 → 7.38
Rooting depth × 0.5	+110.7 ± 12.0	−16.4	0	0.73 → 7.40
Rooting depth × 1.5	+148.0 ± 15.0	+11.8	1	0.73 → 7.31
LAI × 0.7	+167.3 ± 11.2	+26.3	0	0.73 → 7.27
LAI × 1.3	+108.6 ± 12.9	−17.9	1	0.73 → 7.39
*V*_cmax25_ × 0.7	+109.4 ± 8.7	−17.4	0	0.73 → 7.33
*V*_cmax25_ × 1.3	+148.6 ± 16.0	+12.2	1	0.73 → 7.38
Stomatal slope g_1_ = 3 kPa^0.5^	+143.0 ± 12.2	+8.1	0	0.73 → 7.35
Stomatal slope g_1_ = 5 kPa^0.5^	+123.2 ± 12.7	−7.0	0	0.73 → 7.38
Turgor loss point − 0.5 MPa	+134.2 ± 12.4	+1.3	0	0.73 → 7.37
Turgor loss point + 0.5 MPa	+130.1 ± 12.6	−1.7	0	0.73 → 7.38

**Table A9:** SSP1-2.6 and SSP5-8.5 with year-round soil water (five GCMs, 11-year windows, 27 cells per GCM; *A*_season_ and its changes in gC m^−2^ per season, means across cells). The standard errors of the ensemble means across the 135 cells are 3.1 (SSP1-2.6 net change), 6.4 (SSP5-8.5 net change), 6.6 (contrast from own baselines), and 5.7 gC m^−2^ (contrast on the 2070s levels). The contrast on the 2070s levels is SSP5-8.5 minus SSP1-2.6 in 2070–2080, which equals the difference between the two changes measured from any common baseline; the contrast from own baselines is the difference between the two net changes. The approximate 95% intervals (Section 4.9) are +1 to +67 gC m^−2^ for the SSP1-2.6 net change (from the five GCM means; the crossed random-effects interval is +11 to +60 gC m^−2^) and +34 to +148 gC m^−2^ for the contrast on the 2070s levels (crossed random effects). Because of rounding, differences between the rounded levels may differ by 0.1 from the net changes.

GCM	SSP1-2.6, 2020–2030 → 2070–2080	SSP1-2.6 Net Change	SSP5-8.5 Net Change	Contrast, Own Baselines	Contrast, 2070s Levels	Days Above *T*_crit_, SSP1-2.6
GFDL-ESM4	412.9 → 478.7	+65.8	+117.3	+51.4	+80.4	1.6 → 2.0
IPSL-CM6A-LR	404.4 → 426.9	+22.5	+89.4	+66.9	+101.9	1.8 → 3.6
MPI-ESM1-2-HR	386.5 → 402.1	+15.6	+84.3	+68.7	+101.8	1.4 → 1.6
MRI-ESM2-0	415.1 → 472.9	+57.8	+108.3	+50.5	+67.5	2.1 → 2.2
UKESM1-0-LL	430.4 → 436.7	+6.3	+133.4	+127.1	+121.0	2.2 → 4.7
Ensemble	409.8 → 443.5	+33.6	+106.5	+72.9	+94.5	1.8 → 2.8

**Table A10:** Days above the photosystem II threshold per season with thresholds other than the assigned ones (SSP5-8.5, year-round soil water, five GCMs, 11-year windows; 2020–2030 → 2070–2080, means across cells). The assigned thresholds are 46 °C for the conifers (85 cells), 45 °C for *Quercus coccifera* (15 cells), and 44 °C for the deciduous species (35 cells); the counts were made at 1–3 °C below and above them (—, not computed). The last column applies one threshold to all species.

Threshold (°C)	Conifers	*Quercus coccifera*	Deciduous Species	All Cells, Common Threshold
As assigned (46, 45, 44)	0.00 → 2.38	1.15 → 20.79	5.10 → 32.29	1.45 → 12.18
41	—	—	22.90 → 65.47	—
42	—	13.21 → 54.18	15.06 → 54.18	—
43	0.52 → 13.34	7.25 → 41.91	9.32 → 42.86	3.55 → 24.17
44	0.13 → 8.15	3.21 → 30.44	5.10 → 32.29	1.76 → 16.88
45	0.02 → 4.72	1.15 → 20.79	2.56 → 22.94	0.80 → 11.23
46	0.00 → 2.38	0.38 → 12.99	1.05 → 15.38	0.31 → 6.93
47	0.00 → 1.10	0.07 → 7.43	0.37 → 9.60	0.10 → 4.01
48	0.00 → 0.47	0.01 → 3.85	—	—
49	0.00 → 0.27	—	—	—

**Table A11:** The modeled leaf of *Pinus brutia* at fixed conditions (*V*_cmax25_ = 40 μmol m^−2^ s^−1^; intercellular CO_2_ 0.766 C_a_, the ratio of the stomatal model at a vapor pressure deficit of 1.5 kPa; no water stress) at C_a_ of 431.8 and 801.7 μmol mol^−1^, the values of 2020–2030 and 2070–2080 under SSP5-8.5. ET, limited by electron transport (RuBP regeneration); Rubisco, limited by carboxylation capacity. *A* is net assimilation (μmol m^−2^ s^−1^), 0.5*V*_o_/*V*_c_ the photorespiratory CO_2_ release per carboxylation, and the photons are those used per gross CO_2_ fixed. Under the same conditions, the stimulation of *Quercus cerris* and *Q. coccifera* was within 2.0 percentage points of that of *P. brutia* at 20–25 °C, +30.2% and +34.7% at 30 °C, and +102.7% and +103.4% at 35 °C in full light. Net assimilation is zero at 43.3 °C (431.8 μmol mol^−1^) and 45.7 °C (801.7 μmol mol^−1^) at C_i_ = C_a_ in full light, for every species.

*T*_leaf_ (°C)	PAR (μmol m^−2^ s^−1^)	Limitation	*A*, 431.8	*A*, 801.7	Stimulation (%)	0.5*V*_o_/*V*_c_	Photons per CO_2_
20	1800	ET → ET	6.36	7.48	+17.6	0.107 → 0.056	12.6 → 10.8
20	300	ET → ET	5.56	6.54	+17.6	0.106 → 0.056	12.6 → 10.8
25	1800	ET → ET	7.58	9.31	+22.8	0.137 → 0.072	13.8 → 11.4
25	300	ET → ET	6.26	7.69	+22.9	0.136 → 0.072	13.8 → 11.4
30	1800	ET → ET	8.33	10.78	+29.4	0.174 → 0.093	15.4 → 12.1
30	300	ET → ET	6.43	8.35	+29.9	0.172 → 0.092	15.3 → 12.1
35	1800	Rubisco → Rubisco	4.95	10.02	+102.2	0.217 → 0.117	17.4 → 13.0
35	300	Rubisco → ET	4.95	7.87	+58.9	0.217 → 0.116	17.4 → 13.0

**Table A12:** Exposure of the leaves to the photosystem II thresholds from the hourly leaf temperatures (set (ix): SSP5-8.5, year-round soil water, five GCMs, 11-year windows, 135 cells). Each cell gives the mean per season in 2020–2030 and 2070–2080. A season has 3252 lit hours for the conifers, 2892 for *Quercus coccifera*, and 2184 for the deciduous species. The hours above a threshold come in pairs because the sub-daily forcing is symmetric about noon (Equation (A12)). *T*_crit_ + 5.32 and + 6.50 °C correspond to the mean differences between *T*_50_ and *T*_crit_ in the paired measurements of the source data [12]. Expected days with a threshold varying among leaves: Σ_d_ Φ[(*T*_d_ − *T*_crit_)/σ] over the days of the season (Section 4.7). With acclimation, the thresholds of 2070–2080 were raised by 0.2 or 0.4 times the warming of the growing season of each cell; the values for 2020–2030 are those at the assigned thresholds.

Quantity per Season	Conifers	*Quercus coccifera*	Deciduous Species	All Cells
Days with a peak at or above *T*_crit_	0.0 → 2.4	1.2 → 20.8	5.1 → 32.3	1.5 → 12.2
Seasons reaching *T*_crit_ at least once (%)	0.2 → 33.6	28.5 → 92.7	47.8 → 97.1	15.7 → 56.6
Hours at or above *T*_crit_	0.0 → 7.2	2.8 → 68.0	13.9 → 113.1	3.9 → 41.4
Hours above *T*_crit_ per day above it	2.0 → 3.0	2.5 → 3.3	2.7 → 3.5	2.7 → 3.4
Days with 2 h above *T*_crit_	0.00 → 1.37	0.88 → 9.76	3.32 → 12.90	0.96 → 5.29
Days with 4 h above *T*_crit_	0.00 → 0.82	0.27 → 9.00	1.70 → 14.74	0.47 → 5.34
Days with 6 h above *T*_crit_	0.00 → 0.19	0.00 → 1.93	0.07 → 4.44	0.02 → 1.48
Days with 8 h above *T*_crit_	0.00 → 0.00	0.00 → 0.11	0.00 → 0.21	0.00 → 0.07
Longest exceedance in any season (h)	2 → 8	4 → 8	6 → 8	6 → 8
Degree-hours above *T*_crit_ (°C h)	0.0 → 9.5	2.2 → 112.1	16.2 → 238.3	4.5 → 80.2
Lit hours with leaf temperature ≥ 40 °C	20.7 → 160.7	111.7 → 361.2	112.8 → 343.5	54.7 → 230.4
Lit hours with air temperature ≥ 40 °C	0.9 → 49.9	1.3 → 63.0	0.7 → 41.0	0.9 → 49.0
Days with a peak at or above *T*_crit_ + 5.32 °C	0.00 → 0.05	0.00 → 0.52	0.01 → 2.48	0.00 → 0.73
Days with a peak at or above *T*_crit_ + 6.50 °C	0.00 → 0.01	0.00 → 0.26	0.00 → 0.97	0.00 → 0.28
Expected days, threshold varying among leaves, σ = 2.38 °C	0.3 → 5.1	4.6 → 25.5	9.1 → 35.4	3.0 → 15.2
Expected days, threshold varying among leaves, σ = 2.87 °C	0.5 → 6.3	6.1 → 27.2	10.6 → 36.6	3.8 → 16.5
Days, all thresholds 2.38 °C lower	0.3 → 9.9	9.2 → 46.3	17.8 → 58.5	5.8 → 26.5
Days, all thresholds 2.87 °C lower	0.4 → 12.5	12.2 → 52.5	21.7 → 64.0	7.3 → 30.3
Days, all thresholds 2.38 °C higher	0.0 → 0.4	0.0 → 5.9	0.7 → 13.0	0.2 → 4.3
Days, all thresholds 2.87 °C higher	0.0 → 0.3	0.0 → 4.2	0.4 → 10.3	0.1 → 3.3
Days, thresholds acclimated by 0.2 °C per °C	0.0 → 1.4	1.2 → 15.0	5.1 → 25.1	1.5 → 9.0
Days, thresholds acclimated by 0.4 °C per °C	0.0 → 0.8	1.2 → 10.5	5.1 → 18.9	1.5 → 6.5

**Table A13:** The Rubisco constants on a chloroplast basis and all five changes in Section 2.12 together, with the five GCMs (SSP5-8.5, 135 cells, 2023–2027 and 2073–2077; gC m^−2^ per season unless stated; the differences are means ± SE across cells). Reference: The runs with the constants of Table A15, with year-round soil water (the five-year chains of Section 2.10) or with the seasonal reset (Section 2.9, Table A4). The last-in terms are those of Section 2.10; with the seasonal reset, the terms are those of Equation (1), with the channel held at the paired baseline year. The photorespiratory CO_2_ release caused by the warming is that of the run with all channels changed minus that of the run with the warming channel at its baseline. All five changes: The PAR curve over the astronomical day, the transpiration with the boundary-layer conductance, the chloroplast-basis constants, the scale of the vulnerability curve with 50% loss at *P*_50_, and the shape of the measured curve of *Pinus nigra* (*c* = 4.40).

Quantity	Reference	Variant	Difference
Rubisco constants on a chloroplast basis, year-round soil water			
Net change, 2023–2027 to 2073–2077	+109.1	+112.8	+3.7 ± 0.4
Last-in CO_2_ term, 2073–2077	+212.8	+215.9	+3.1 ± 0.3
Last-in warming term, 2073–2077	−67.1	−65.5	+1.6 ± 0.2
*A*_season_, 2023–2027	431.7	435.2	+3.5 ± 0.7
*A*_season_, 2073–2077	540.7	548.0	+7.2 ± 1.0
Photorespiratory CO_2_ release, 2023–2027	73.7	65.0	−8.7 ± 0.4
Photorespiratory CO_2_ release, 2073–2077	55.2	46.2	−9.0 ± 0.4
Days above *T*_crit_, 2073–2077	12.03	12.02	−0.01 ± 0.00
Rubisco constants on a chloroplast basis, seasonal reset			
Net change, 2023–2027 to 2073–2077	+131.3	+136.0	+4.7 ± 0.4
CO_2_ term, 2073–2077	+219.4	+223.3	+4.0 ± 0.4
Warming term, 2073–2077	−63.8	−61.9	+1.9 ± 0.2
Photorespiratory CO_2_ release, 2073–2077	58.9	49.2	−9.7 ± 0.3
Photorespiratory CO_2_ release caused by the warming, 2073–2077	+4.3	+1.8	−2.5 ± 0.1
All five changes together, year-round soil water			
Net change, 2023–2027 to 2073–2077	+109.1	+115.6	+6.5 ± 1.0
*A*_season_, 2023–2027	431.7	476.3	+44.6 ± 2.0
*A*_season_, 2073–2077	540.7	591.8	+51.1 ± 2.3
Photorespiratory CO_2_ release, 2073–2077	55.2	48.4	−6.8 ± 0.3
Transpiration, 2073–2077 (mm)	341.8	348.0	+6.2 ± 0.5
Days above *T*_crit_, 2073–2077	12.03	12.15	+0.11 ± 0.02
Cells with a decline	10	11	

## Appendix C. Climate of the Growing Season at the Sites

Table A14 gives the climate of the conifer growing season (days 60–330 of the year) at the six sites in the two windows, as the model received it from the ISIMIP3b forcing (Section 4.5).

**Table A14:** The climate of the conifer growing season (days 60–330) at the six sites: means for 2020–2030 and changes to 2070–2080 (five-GCM means). *T* is the mean of the daily maximum and minimum air temperatures, *T*_max_ is the daily maximum, VPD is the daily vapor pressure deficit, PAR is the daily peak of photosynthetically active radiation, and *P* is precipitation over the season and the year. C_a_ rose from 431.8 to 801.7 μmol mol^−1^ under SSP5-8.5 and from 427.7 to 471.0 μmol mol^−1^ under SSP1-2.6. Among the GCMs, Δ*T* ranged from 1.7 to 4.6 °C and the change in annual precipitation from −24.7% to +3.6% under SSP5-8.5.

Site	*T*, 2020–2030 (°C)	Δ*T* (°C)	Δ*T*_max_ (°C)	ΔVPD (kPa)	ΔPAR (%)	Δ*P*, Season (%)	Δ*P*, Year (%)
SSP5-8.5							
Antalya	22.9	+3.1	+3.3	+0.41	+1.2	−7.5	−10.3
Balıkesir	18.6	+3.1	+3.5	+0.34	+2.4	−15.4	−16.3
Konya	15.5	+3.3	+3.5	+0.37	+1.5	−11.8	−15.1
Eskişehir	16.1	+3.3	+3.8	+0.40	+2.3	−14.0	−13.5
Kahramanmaraş	16.0	+3.5	+3.7	+0.38	+1.0	−7.3	−14.9
Rize	18.9	+2.9	+3.0	+0.18	+1.0	−2.5	−2.3
SSP1-2.6							
Antalya	23.1	+0.4	+0.5	+0.03	+0.9	+4.2	+4.9
Balıkesir	18.9	+0.5	+0.6	+0.03	+1.4	−1.8	−1.6
Konya	15.6	+0.5	+0.6	+0.03	+0.9	+8.2	+4.5
Eskişehir	16.3	+0.5	+0.5	+0.02	+1.4	+5.3	+4.4
Kahramanmaraş	16.1	+0.6	+0.7	+0.05	+1.1	+0.4	+1.7
Rize	19.0	+0.6	+0.6	+0.03	+1.9	+1.1	+0.9

## Appendix D. Model Equations and Parameters

The equations below are those of the reported implementation (Section 4.8); Table A15 gives the parameters common to all species and Table A16 the species traits. Leaf temperature *T* is in °C and *T*_k_ in K, CO_2_ mole fractions are in μmol mol^−1^, and O_2_ is in mmol mol^−1^. In the Rubisco-limited state, at a capacity scale ξ, carboxylation, oxygenation, and net assimilation are

*V*_c_ = ξ *V*_cmax_(*T*) *C*_m_/[*C*_m_ + *K*_c_(*T*)(1 + *O*/*K*_o_(*T*))] (A1)

*V*_o_ = 2Γ*(*T*) *V*_c_/*C*_m_, *A* = *V*_c_ − 0.5*V*_o_ − *R*_d_(*T*) = *g*_m_(*T*)(*C*_i_ − *C*_m_) (A2)

where *C*_m_ and *C*_i_ are the chloroplast and intercellular CO_2_ mole fractions and *g*_m_ is the mesophyll conductance. The demand for ATP and NADPH, counted per carboxylation and per oxygenation including the photorespiratory cycle, and the linear (LEF) and cyclic (CEF) electron flows that meet it, with *h* = 4.67 H^+^ per ATP [69], are

ATP = 3*V*_c_ + 3.5*V*_o_, NADPH = 2*V*_c_ + 2.5*V*_o_ (A3)

*J*_LEF_ = 2 NADPH, *J*_CEF_ = (*h*/2) max(ATP − 3*J*_LEF_/*h*, 0), *J*_dem_ = *J*_LEF_ + *J*_CEF_ (A4)

Electron transport is supplied according to the non-rectangular hyperbola

*J* = {*I*_e_ + *J*_max_ − [(*I*_e_ + *J*_max_)^2^ − 4θ*I*_e_*J*_max_]^1/2^}/(2θ), *I*_e_ = α_L_(1 − *f*) PAR/*p*_e_ (A5)

where PAR is the incident photosynthetically active radiation and *p*_e_ = (2*J*_LEF_ + *J*_CEF_)/(*J*_LEF_ + *J*_CEF_) is the number of photons per electron. The capacity scale ξ equals the water-stress multiplier β (Equation (A10)) when *J*_dem_ ≤ *J*; otherwise ξ < β is solved so that *J*_dem_ = *J* (electron-transport limitation). For C_3_ leaves, this is equivalent to

*A* = *J*(*C*_m_ − Γ*)/(5.005*C*_m_ + 11.345Γ*) − *R*_d_ (A6)

against 4*C*_m_ + 8Γ* in the FvCB expression [67,68], so the budget spends 28–30% more electrons per CO_2_ fixed. Triose phosphate use and the light inhibition of day respiration are not represented. The temperature responses are the Arrhenius function and its peaked form,

*f*_A_(*T*) = exp[*H*_a_(*T*_k_ − 298.15)/(298.15 *R T*_k_)] (A7)

*f*_P_(*T*) = *f*_A_(*T*) [1 + exp((298.15 Δ*S* − *H*_d_)/(298.15 *R*))]/[1 + exp((*T*_k_ Δ*S* − *H*_d_)/(*R T*_k_))] (A8)

with *R* = 8.314 J mol^−1^ K^−1^ and the parameters in Table A15. Stomatal conductance to water vapor follows the optimal stomatal model [71] and is coupled to the CO_2_ supply,

*g*_s_ = *g*_0_ + 1.6(1 + *g*_1_/*D*_leaf_^1/2^) max(*A*, 0)/*C*_a_, *A* = (*g*_s_/1.6)(*C*_a_ − *C*_i_) (A9)

where *D*_leaf_ is the leaf-to-air vapor pressure deficit (at least 0.05 kPa). Water stress acts as a ceiling on the carboxylation capacity (ξ ≤ β), with

β = 1 for ψ_l_ ≥ ψ_on_; β = (ψ_l_ − ψ_TLP_)/(ψ_on_ − ψ_TLP_) for ψ_TLP_ < ψ_l_ < ψ_on_; β = 0 for ψ_l_ ≤ ψ_TLP_ (A10)

where ψ_l_ is the leaf water potential, ψ_TLP_ is the turgor loss point, and ψ_on_ = 0.5ψ_TLP_ is the onset of water stress; β is bounded below by 10^−4^, so that at the turgor loss point net assimilation falls to about −*R*_d_ and *g*_s_ to *g*_0_. Leaf temperature follows the linearized Penman equation [73],

*T*_leaf_ − *T*_air_ = (*R*_n_ − λ*g*_v_*D*_air_/*P*)/(*c*_p_*g*_Hr_ + λ*g*_v_*s*′/*P*) (A11)

with *R*_n_ = *a*_sw_SW + ε_L_(ε_sky_ − 1)σ*T*_air,k_^4^ the isothermal net radiation of one leaf side (SW = PAR/(4.57 × 0.5) W m^−2^), *g*_v_ = (1/*g*_s_ + 1/*g*_b_)^−1^, *g*_Hr_ = *g*_b_/1.08 + 4ε_L_σ*T*_air,k_^3^/*c*_p_, *s*′ the slope of the saturation vapor pressure curve, *P* is the air pressure, and *D*_air_ is the vapor pressure deficit of the air; *D*_leaf_ is then *e*_s_(*T*_leaf_) minus the vapor pressure of the air. The daily forcing (maximum and minimum temperature, relative humidity, shortwave radiation, and precipitation) is distributed over 24 hourly steps, each the mean of 12 sub-samples, by

*x* = sin[π(*h* − 6)/12], *T*_air_ = *T*_min_ + (*T*_max_ − *T*_min_)*x*^0.8^, *D*_air_ = *D*_d_(0.25 + 0.75*x*), PAR = PAR_pk_*x* (A12)

for 6 ≤ *h* ≤ 18 h and *x* = 0 otherwise, where *D*_d_ is the daily vapor pressure deficit from the mean of the saturation vapor pressures at the daily extremes and the relative humidity, PAR_pk_ = (π/2)PAR_day_/*N*, and *N* is the astronomical day length. The soil–plant hydraulics follow Photo3 [103]: with relative soil moisture *s*,

ψ_s_ = ψ_sat_*s*^−*b*^, *K* = *K*_s_*s*^2*b*+3^ (A13)

*g*_sr_ = *K*(*s*)(RAI *s*^−*d*^)^1/2^/(π ρ_w_ *G Z*_r_), *g*_p_ = *k*_max_ exp[−(−ψ_l_/|*P*_50_|)*^c^*] (A14)

where ψ_s_ is the soil water potential, *K* is the hydraulic conductivity, *g*_sr_ is the soil–root and *g*_p_ the plant conductance (both in mmol m^−2^ s^−1^ MPa^−1^ after conversion), RAI is the root area index, ρ_w_ is the density of water, *G* = 9.81 m s^−2^, and *Z*_r_ is the rooting depth; with the scale |*P*_50_|, 37% of *k*_max_ remains at *P*_50_ (Section 4.2). Leaf transpiration and leaf water potential satisfy

*E* = *g*_s_*D*_leaf_/*P* = (1/*g*_sr_ + 1/*g*_p_)^−1^(ψ_s_ − ψ_l_) (A15)

and the soil water store is updated once per day,

*nZ*_r_ Δ*s* = Pr − Ē LAI − *K*(*s*) − *E*_max_(*s* − *s*_h_)/(1 − *s*_h_) (A16)

where Pr is precipitation, Ē is the 24 h mean leaf transpiration (mm d^−1^), and the last two terms are drainage and soil evaporation, with *s* kept between *s*_h_ and 1. With the seasonal reset, *s* = 0.60 at the start of each season; with year-round soil water, the store continues between seasons with *E* = 0. A day counts as a day above *T*_crit_ when the hourly leaf temperature of any lit step reaches *T*_crit_. Two simplifications follow from these equations. The radiation curve of Equation (A12) spans a fixed 12 h, whereas its peak is computed for the astronomical day length, so the leaf receives 12/*N* of the daily PAR of the forcing: 80% at the summer solstice and 88–96% on average over the growing seasons at these latitudes. The transpiration charged to the soil (Equation (A15)) uses the stomatal conductance alone, whereas the latent heat of the energy balance (Equation (A11)) uses the stomatal and boundary-layer conductances in series, so the soil is charged a factor 1 + *g*_s_/*g*_b_ more water, about 5% for needles and 13% for broadleaves at *g*_s_ = 0.15 mol m^−2^ s^−1^. A third simplification concerns the Rubisco constants of Table A15. They were estimated on an intercellular basis, with an infinite mesophyll conductance [37], but are applied at *C*_m_. With the chloroplast-basis constants at 25 °C, Γ* = 37.43 μmol mol^−1^, *K*_c_ = 272.38 μmol mol^−1^, and *K*_o_ = 165.82 mmol mol^−1^, with activation energies of 24.46, 80.99, and 23.72 kJ mol^−1^ [40], the photorespiratory CO_2_ release per carboxylation of the leaf of Table A11 at 431.8 μmol mol^−1^ was 0.12 instead of 0.14 at 25 °C and 0.16 instead of 0.22 at 35 °C, and its CO_2_ stimulation +20%, +23%, and +90% instead of +23%, +29%, and +102% at 25, 30, and 35 °C. In absolute terms, the increase in net assimilation in full light was smaller at 25 and 30 °C (1.59 and 2.17 instead of 1.73 and 2.45 μmol m^−2^ s^−1^) but larger at 35 °C, where the leaf was Rubisco-limited (5.33 instead of 5.07 μmol m^−2^ s^−1^). The effects of the three simplifications over the season are given in Section 2.12.

**Table A15:** Parameters common to all species. Activation and deactivation energies (*H*_a_, *H*_d_) are in kJ mol^−1^ and entropy terms (Δ*S*) in kJ mol^−1^ K^−1^; values at 25 °C precede them.

Parameter	Symbol	Value	Unit	Source
Photosynthesis				
O_2_ mole fraction	*O*	210	mmol mol^−1^	—
Michaelis constant for CO_2_ at 25 °C; *H*_a_	*K*_c_	404.9; 79.43	μmol mol^−1^	[37]
Michaelis constant for O_2_ at 25 °C; *H*_a_	*K*_o_	278.4; 36.38	mmol mol^−1^	[37]
CO_2_ compensation point without *R*_d_ at 25 °C; *H*_a_	Γ*	42.75; 37.83	μmol mol^−1^	[37]
Temperature response of *V*_cmax_	*H*_a_; *H*_d_; Δ*S*	65.33; 200; 0.650	—	*H*_a_ [37]; others assumed
Temperature response of *J*_max_	*H*_a_; *H*_d_; Δ*S*	43.54; 200; 0.640	—	Assumed
Ratio of *J*_max25_ to *V*_cmax25_	—	1.6 (*Q. coccifera* 1.73)	—	Assumed (*Q. coccifera*: Table A16)
Day respiration at 25 °C; *H*_a_	*R*_d25_	0.015 *V*_cmax25_; 46.39	μmol m^−2^ s^−1^	*H*_a_ [37]; ratio assumed
Mesophyll conductance at 25 °C; *H*_a_; *H*_d_; Δ*S*	*g*_m25_	0.40; 49.6; 437.4; 1.4	mol m^−2^ s^−1^	Response [40]; *g*_m25_ assumed
Curvature of the light response	θ	0.70	—	[68]
Leaf absorptance for PAR	α_L_	0.85	—	[68]
Spectral correction	*f*	0.15	—	[68,70]
Protons per ATP (c_14_ ATP synthase)	*h*	4.67	—	[69]
Stomata and water stress				
Residual conductance	*g*_0_	0.01	mol m^−2^ s^−1^	Assumed
Slope of the stomatal model	*g*_1_	4.0	kPa^0^·^5^	Assumed (Section 3.4)
Onset of water stress	ψ_on_	0.5 ψ_TLP_	MPa	Assumed
Energy balance				
Shortwave absorptance	*a*_sw_	0.50	—	Assumed (Section 2.5)
Leaf and sky emissivity	ε_L_; ε_sky_	0.97; 0.80	—	ε_L_ [73]; ε_sky_ assumed
Boundary-layer conductance (conifer, sclerophyll, deciduous)	*g*_b_	3.0, 1.6, 1.2	mol m^−2^ s^−1^	Assumed (Section 2.5)
Latent heat; heat capacity of air; air pressure	λ; *c*_p_; *P*	44 kJ mol^−1^; 29.3 J mol^−1^ K^−1^; 101.3 kPa	—	[73]
Soil (sandy loam) and roots				
Saturated hydraulic conductivity	*K*_s_	80	cm d^−1^	[103]
Soil water potential at saturation	ψ_sat_	−0.7	kPa	[103]
Retention exponent; porosity; hygroscopic point	*b*; *n*; *s*_h_	4.90; 0.43; 0.14	—	[103]
Root area index; exponent	RAI; *d*	5.6; 8	m^2^ m^−2^; —	[103] (RAI of crops)
Maximum soil evaporation	*E*_max_	1.0	mm d^−1^	Assumed
Shape of the vulnerability curve	*c*	2.0 (*P. nigra* 2.21)	—	Assumed; Table A16
Thresholds, seasons and steps				
Critical temperature of photosystem II (conifer, sclerophyll, deciduous)	*T*_crit_	46, 45, 44	°C	44 °C [12]; others assumed
Growing season (conifer, sclerophyll, deciduous)	—	60–330, 60–300, 105–286	day of year	Assumed
Relative soil moisture at the start of each season (seasonal reset)	*s*	0.60	—	Assumed
Sub-daily steps	—	24	d^−1^	—

**Table A16:** Species traits. M, measured on the species; S, surrogate value of a congener; A, assumed. *J*_max25_ = 1.6 *V*_cmax25_ (*Quercus coccifera* 131.7 μmol m^−2^ s^−1^), *R*_d25_ = 0.015 *V*_cmax25_, and *g*_m25_ = 0.40 mol m^−2^ s^−1^ for all species.

Species	*P*_50_ (MPa)	ψ_TLP_ (MPa)	*k*_max_ (mmol m^−2^ s^−1^ MPa^−1^)	*Z*_r_ (m)	LAI (m^2^ m^−2^)	*V*_cmax25_ (μmol m^−2^ s^−1^)
*Pinus brutia*	−3.57 ^S^	−2.6 ^A^	5.5 ^A^	1.0 ^A^	2.0 ^A^	40.0 ^A^
*Pinus nigra* subsp. *pallasiana*	−2.76 ^S^	−2.5 ^A^	6.5 ^A^	1.2 ^A^	2.5 ^A^	42.0 ^A^
*Cedrus libani*	−6.95 ^M^	−2.8 ^A^	5.0 ^A^	1.5 ^A^	2.1 ^A^	44.0 ^A^
*Juniperus excelsa*	−8.70 ^S^	−3.2 ^A^	3.5 ^A^	2.0 ^A^	1.5 ^A^	38.0 ^A^
*Picea orientalis*	−3.63 ^S^	−2.3 ^A^	7.0 ^A^	0.8 ^A^	5.0 ^A^	40.0 ^A^
*Quercus coccifera*	−7.13 ^S^	−2.6 ^A^	7.0 ^A^	2.5 ^A^	3.5 ^A^	76.2 ^S^
*Quercus cerris*	−4.90 ^M^	−1.9 ^S^	8.0 ^A^	2.0 ^A^	4.0 ^A^	55.0 ^A^
*Fagus orientalis*	−3.20 ^A^	−2.6 ^S^	9.0 ^A^	1.0 ^A^	5.5 ^A^	50.0 ^A^

*P*_50_: *Pinus brutia*, *P. halepensis* [104]; *P. nigra* subsp. *pallasiana*, *P. nigra* seedlings [105]; *Cedrus libani*, midpoint of −6.4 to −7.5 MPa reported for *C. libani* and *C. brevifolia* [106]; *Juniperus excelsa*, *J. seravschanica*, which Larter et al. [107] treat as *J. excelsa* subsp. *polycarpos*, whereas the Anatolian stands belong to subsp. *excelsa*; *Picea orientalis*, *P. abies* [108]; *Quercus cerris*, the water potential at 40% loss of petiole conductivity in seedlings [109]; *Q. coccifera*, *Q. ilex* [16] (Table A1 also uses −3.88 [14] and −4.66 MPa [15], and −6.96 MPa is reported for *Q. coccifera* itself [15]); *Fagus orientalis*, assumed slightly more negative than the provenance means of *F. sylvatica*, from −2.70 to −3.12 MPa, which did not differ among provenances [110]. Turgor loss point: *F. orientalis*, *F. sylvatica* [111]; *Q. cerris*, *Q. pubescens*, whose treatment means were from −1.57 to −2.10 MPa [12,102] (Appendix A). *V*_cmax25_ of *Q. coccifera*: *Q. ilex* [112], the seasonal maximum of current-year leaves in the control plot from A–*C*_c_ curves fitted with the chloroplast-basis constants [40]; with these constants, the net change of *Q. coccifera* was +112.3 instead of +109.6 gC m^−2^ (year-round soil water, five GCMs; Section 2.12). LAI of *C. libani*: assumed, within the range of 1.04–3.22 measured in four stands [113]. The rooting depths lie below the biome means of the maximum rooting depth, 2.9 m for temperate deciduous forest, 3.9 m for temperate coniferous forest, and 5.2 m for sclerophyllous shrubland and forest [114]. The vulnerability curve has the shape *c* = 2.0 for all species except *P. nigra*, for which the slope of the logistic curve fitted by Froux et al. [105] (2.21) was used as the shape parameter; the two are not equivalent, and a Weibull curve with the slope of the fitted curve at *P*_50_ has *c* = 4.40, so the modeled curve of *P. nigra* is shallower than the measured one; with *c* = 4.40, the net change of *P. nigra* was 0.1 gC m^−2^ larger (Section 2.12). All other values are assumed.
